# Epigenetic Heterogeneity of B-Cell Lymphoma: DNA Methylation, Gene Expression and Chromatin States

**DOI:** 10.3390/genes6030812

**Published:** 2015-09-07

**Authors:** Lydia Hopp, Henry Löffler-Wirth, Hans Binder

**Affiliations:** Interdisciplinary Centre for Bioinformatics, Universität Leipzig, Härtelstr. 16–18, 04107 Leipzig, Germany; E-Mail: wirth@izbi.uni-leipzig.de

**Keywords:** germinal center, epigenetic reprogramming, stemness, promoter methylation, gene expression, machine learning, high dimensional data portraying

## Abstract

Mature B-cell lymphoma is a clinically and biologically highly diverse disease. Its diagnosis and prognosis is a challenge due to its molecular heterogeneity and diverse regimes of biological dysfunctions, which are partly driven by epigenetic mechanisms. We here present an integrative analysis of DNA methylation and gene expression data of several lymphoma subtypes. Our study confirms previous results about the role of stemness genes during development and maturation of B-cells and their dysfunction in lymphoma locking in more proliferative or immune-reactive states referring to B-cell functionalities in the dark and light zone of the germinal center and also in plasma cells. These dysfunctions are governed by widespread epigenetic effects altering the promoter methylation of the involved genes, their activity status as moderated by histone modifications and also by chromatin remodeling. We identified four groups of genes showing characteristic expression and methylation signatures among Burkitt’s lymphoma, diffuse large B cell lymphoma, follicular lymphoma and multiple myeloma. These signatures are associated with epigenetic effects such as remodeling from transcriptionally inactive into active chromatin states, differential promoter methylation and the enrichment of targets of transcription factors such as *EZH2* and *SUZ12*.

## 1. Introduction

**Figure 1:**
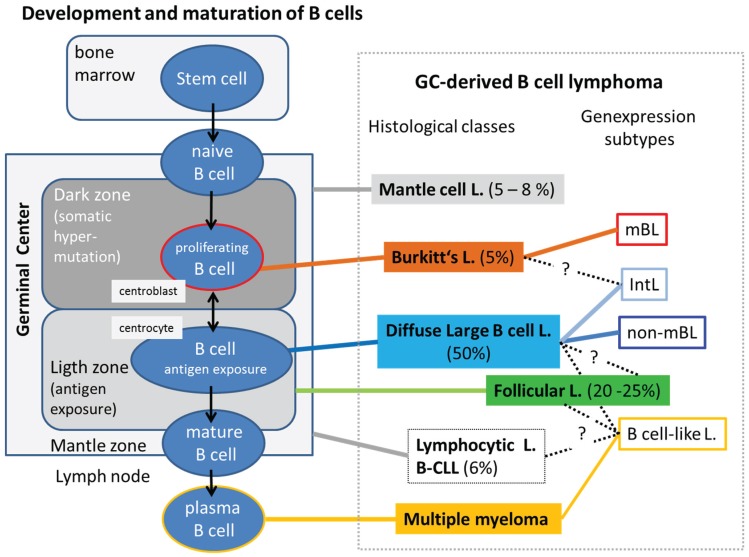
Developmental and maturation stages of B-cells provide a wide spectrum of cell-of-origin- and micro-environmental conditions for different histological classes of B-cell lymphoma. In this study, we focus on germinal center derived B-cell lymphoma and multiple myeloma. Molecular gene expression subtypes were taken from [4,5,6]. Their relation to the histological classes is partly unclear mainly due to the absence of clear-cut borderlines between the molecular and histological signatures and because of transformations between the classes. Incidence rates in percentage of all B-cell lymphoma were taken from http://www.cancerresearchuk.org/cancer-info/cancerstats/types/nhl [11].

B-cells are lymphocytes that are an essential component of the adaptive immune system. Immature “naïve” B-cells are produced in the bone marrow which then migrate to germinal centers (GC) where they differentiate into mature B-lymphocytes (Figure 1). These GC are central to the formation of B-cell-mediated immunity: B-cells undergo immunoglobulin somatic hypermutation and clonal expansion via intense proliferation in the dark zone and subsequent antigen exposure and selection in the light zone of the GC to generate ultimately long-lived memory B-cells and terminally differentiated plasma cells expressing high-affinity antibodies. B-cell development is a multistep process sustained by a highly coordinated transcriptional network modulated by epigenetic mechanisms, including DNA methylation and histone modifications to promote lineage commitment, to define and sustain cell identity and establish heritable cell-type- and stage-specific gene expression profiles [1].

Dysfunction of epigenetic regulation represents a common and important feature of B-cell lymphomas. Available evidence suggests that different diseases arise from oncogenic B-cell clones at a distinct stage of differentiation, ranging from naïve B (NB) cells to plasma cells. These tumors of the lymphoid tissues represent one of the most heterogeneous malignancies owing to the wide spectrum of types of B-cells from which they can arise and also due to the heterogeneous microenvironment in the lymphatic organs providing a multitude of different niches for tumor progression. Many B-cell malignancies derive from germinal center B-cells, most likely because of the high proliferation rate of these cells and the high activity of mutagenic processes. This category includes diffuse large B-cell lymphomas (DLBCL), follicular lymphomas (FL), Burkitt lymphomas (BL) and mantle cell lymphoma (MCL). Mature B-cell malignancies in addition include leukemias derived from B-cells that have passed through the GC such as B-cell chronic lymphocytic leukemia (B-CLL) which is a stage of small lymphocytic lymphoma. Multiple myeloma (MM) is an incurable B-cell neoplasia arising from malignant plasma cells which originates in illegitimate immunoglobulin heavy chain (IgH) switch recombinations.

Morphologic features of lymphomas resemble lymphocytes at distinct differentiation stages serving as basis for their histological classification. Alternatively, the rapidly emerging information obtained from molecular high-throughput gene expression studies created a series of expression-based classification schemes [1,2,3,4,5] which distinguish, for example, molecular Burkitt lymphoma (mBL), non-mBL, intermediate lymphoma (IntL) with an intermediate signature between mBL and non-mBL, and B cell-like lymphoma (BCL) resembling pre- and post-GCB cells [5,6]. Many details of the molecular mechanisms underlying genesis, progression and also mutual transformations across the subtypes are not clear. Changing gene expression signatures are strongly linked to perturbations of epigenetic mechanisms. Understanding molecular mechanisms of lymphoma thus requires a combined view including gene expression, epigenetics and also genetic factors affecting B-cell biology.

B-cells employ epigenetic mechanisms to generate effective memory responses resembling epigenetic reprogramming of stem cells upon cell fate decisions and differentiation. Particularly, the transition from naïve B-cells permits GCB cells to generate the differential response to antigenic challenges and to differentiate toward plasma cell fates. Deregulation of the underlying epigenetic determinants such as DNA methylation [7] and/or chromatin activity states potentially disturbs or even prevents the differentiation of B-cells leading to lymphomas. Altered epigenetic regulation represents a common and important feature of B-cell disorders. For example, GCB cells are prone to instability in their cytosine DNA methylation patterns leading to aberrant methylation patterns in lymphoma, which display variable degrees of epigenetic heterogeneity [2,8,9]. Moreover, polycomb group (PcG) proteins, a subset of histone-modifying enzymes known to be crucial for B-cell maturation and differentiation, play a central role in malignant transformation of B-cells [10]. Genes *de novo* methylated in all lymphoma enrich in polycomb targets and share a similar stem cell-like epigenetic pattern [9].

Our study aims to shed light into the epigenetic mechanisms driving lymphomagenesis and particularly the possible role of chromatin remodeling in the transformations from healthy to malignant B-cells. To this aim, we present an integrative study of gene expression and of DNA methylation data measured in lymphoma cohorts stratified into different lymphoma classes. We previously demonstrated that machine learning using self-organizing maps (SOM) well resolves the molecular landscapes of different cancer types [5,12,13,14]. Our high-dimensional data portraying method is applied here for the first time in an integrative way that combines expression and methylation data.

## 2. Data and Methods

### 2.1. Methylation Data

Microarray-derived DNA methylation data (GoldenGate Methylation Cancer Panel I; Illumina, San Diego, CA) of in total 133 samples obtained from hematological neoplasms and reference systems were taken from [15] in terms of beta values of 1410 CpG’s located in the range of −1500 bp to +500 bp around the transcription start site of 768 genes thus serving as markers for their promoter methylation. The lymphoma samples were classified as diffuse large B-cell lymphoma (DLBCL, 54 samples), molecular Burkitt’s lymphoma (mBL, 18), intermediate lymphoma (IntL, 16), follicular lymphoma (FL, 14) and mantle cell lymphoma (MCL, 10). The data set further contains multiple myeloma (MM, 14), healthy B-cells (5) and germinal center B cells (GCB, 2) as reference. For details of the methylation experiments, the array platform, primary data analysis, sample selection and classification see [15]. Methylation data was given in units of beta values estimating the level of methylation between values of zero (no methylation) and unity (full methylation) for each promoter. Differential methylation defines the difference between beta values of two states, e.g., between lymphoma and healthy B-cells, where hyper- and hypomethylation assigns positive and negative differences (delta beta values), respectively. Integral differential methylation was calculated as mean differential methylation separately averaged over all positive and negative delta beta values. Please take into account that for SOM analysis of differential methylation (DmetSOM, see below) we used centralized methylation data, which are calculated as the difference between the beta value of a given promoter in a given sample and its mean value averaged over all samples studied.

### 2.2. Gene Expression Data

Expression data were taken from the MMML (molecular mechanisms of malignant lymphoma) cohort described in [4] comprising 936 samples. Lymphoma samples were classified into five molecular subtypes as described in [5,6]: molecular BL (mBL, 85 samples), non-molecular Burkitts (non-mBL, 287), intermediate lymphoma (IntL, 307), follicular lymphoma (FL, 121) and B cell like lymphoma (BCL, 64). According to pathological diagnosis, the molecular subtypes refer predominantly to BL (mBL), DLBCL (non-mBL) and MM (BCL). Further, the cohort contains B-cells (17), GCB cells (13), a lymphoma cell line (32) and tonsils (10) as reference. The microarray expression data (Affymetrix HG-U133a) were processed as described previously [5]. The B-cells subsume naïve pre- and mature post-GCB cells which show virtually indistinguishable gene expression patterns. The GCB cells are centroblasts with strongly activated proliferative cellular programs.

### 2.3. High-Dimensional Data Portraying

Preprocessed gene-centric expression and methylation data were clustered using self-organizing map (SOM) machine learning. This method translates the gene data matrix into metagene data of reduced dimensionality. Each metagene (methylation or expression) data were visualized in a sample-specific fashion by arranging the metagenes in a two-dimensional quadratic 50 × 50 grid and by appropriately color coding of the data values. The mosaic images obtained serve as fingerprint portraits of the expression and methylation landscapes of each sample. Class-specific mean portraits were generated by averaging the metagene landscapes of all cases belonging to one class. SOM size and topology was chosen to allow robust identification of expression modules inherent in the data in terms of so-called spot clusters as described in our previous publications [16,17]. Overview spot maps were generated by collecting all hypermethylation or overexpression spots of individual portraits into one map. Three different SOMs were trained using (i) methylation beta values (MetSOM); (ii) centralized beta values with respect to the mean beta of a gene averaged over all samples (DmetSOM); and (iii) centralized log-expression data (DexSOM). Note that genes are arranged differently in each of the SOM trainings. For comparison, we mapped groups of selected genes in each of the SOM maps as described below. For SOM analyses, we used the R-package “oposSOM” which is publically available from the Bioconductor repository [18].

### 2.4. Downstream Bioinformatics Analyses

Downstream analyses comprise the detection of clusters of differentially expressed or methylated genes (so-called spot-cluster detection), significance analysis and diversity analysis by means of correlation nets, which reveal similarity relationships between the samples. For the interpretation of the functional context of groups of genes, we applied gene set enrichment analysis using the gene set enrichment score (GSZ) [19]. The GSZ estimates the degree of reliability that a gene set with reference to a certain biological functionality is related to a collection of genes with unknown functional impact, e.g., derived from differential expression analysis. All downstream methods were described in [16,17], illustrated in a pilot application [12] and implemented in “oposSOM” [18].

## 3. Results

### 3.1. DNA Methylation of Lymphoma

**Figure 2:**
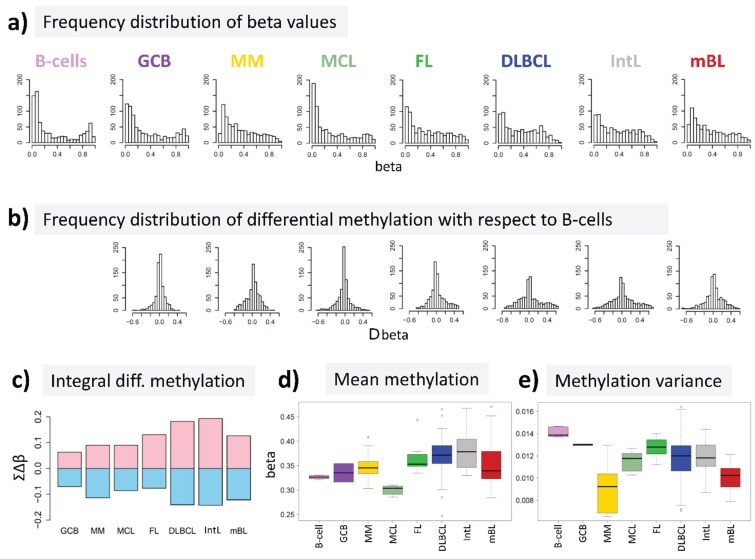
DNA methylation summary characteristics of lymphoma and of healthy B and GCB cells. (**a**) The frequency distribution of the promoter methylation beta values of B-cells shows two maxima referring to almost not- and completely methylated promoters, respectively; The distributions of beta values loose this bimodality to a large degree in lymphoma where weakly and intermediately methylated genes become hyper-methylated and highly methylated genes become hypo-methylated compared with healthy B-cells (**b** + **c**); (**d**) The total methylation level increases and (**e**) the variability of methylation among the genes in each of the samples decreases.

We re-analyzed microarray DNA methylation data published in a previous study on a large number of hematological neoplasms [15] to answer the question, whether DNA methylation differs between B-cells and different histological lymphoma classes. For B-cells we found a bimodal shape of the frequency distribution of beta values among the genes studied with maxima near zero (completely de-methylated CpG sites) and unity (completely methylated, see Figure 2a). The respective distributions of beta values in lymphoma are characterized by a wide loss of this bimodality where especially the fraction of highly methylated genes with beta values near unity markedly decreases. Accordingly, the distributions of beta value alterations of the genes in the different systems compared with their methylation in B-cells were tailed to both, positive and negative values reflecting hypo- and hypermethylation of the respective genes (Figure 2b). The integral hyper- and hypomethylation of all genes considered reveals the progressively increasing disturbance of DNA methylation in lymphoma being largest in DLBCL and IntL, but being relatively small in MM, FL, MCL and also mBL (Figure 2c). This trend agrees with the results of previous studies reporting the gain of epigenetic heterogeneity (in terms of differential methylation with respect to the reference state of healthy B-cells) with progressive aggressiveness of lymphoma being largest in DLBCL [20,21]. Except for MCL, we find a global hypermethylation of the genes in lymphoma compared with B-cells (Figure 2d). On the other hand, the variance of beta values in each of the samples strongly decreases in lymphoma mainly due to the decrease or even loss of bimodality reported above (Figure 2e).

In summary, methylation changes in lymphoma comprise both, hyper- and hypomethylation effects leading to a loss of bimodality of promoter methylation with maxima at low and high beta values and to more balanced methylation landscapes where promoter regions tend to become methylated on intermediate beta-levels.

**Figure 3:**
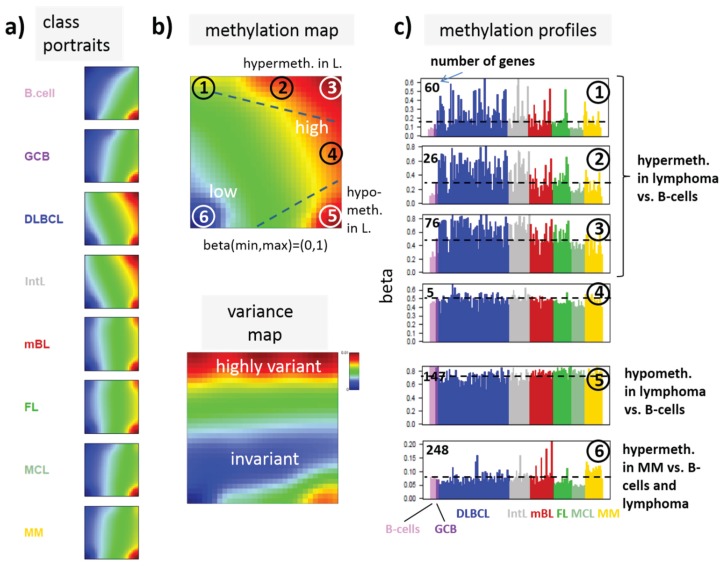
SOM portraying of the DNA methylation landscape of lymphoma (MetSOM). (**a**) SOM portraits of histological lymphoma classes and of controls. Red and blue colors assign regions containing genes with high and low methylation levels, respectively; (**b**) the methylation overview map summarizes regions hypermethylated in any of the classes compared with any other one in red. The methylation variance map identifies regions of highly variable (red) and almost invariant (blue) beta values; (**c**) The methylation profiles show the mean methylation level among the samples of genes taken from the “spot” regions 1–6 assigned in the methylation overview map. Horizontal dashed lines serve as guide for the eye showing the mean methylation level of the respective spot averaged over all samples. Assignments as “hyper-” or “hypomethylated” refer to relative methylations compared with B-cells. Lists of genes in these regions are given in Table S1.

### 3.2. DNA Methylation Portraying

In the next step, SOM data portraying was applied to the methylation data including all lymphoma samples and that of the B and GCB cells serving as reference. The method “projects” the methylation data onto a two-dimensional grid of 50 × 50 pixels. Appropriate color-coding then visualized the methylation landscapes of each sample in terms of its individual methylation portrait shown in Figure S1. We averaged theses portraits taking into account all samples of each class to identify class-specific methylation signatures. Figure 3a shows the gallery of these mean portraits for all classes studied. Red and blue regions in the images refer to genes with high and low methylation levels of the probed CpG regions with beta values near one and zero, respectively. The map can be segmented into regions containing genes hyper- and hypomethylated in lymphoma and a region with almost invariantly methylated genes in between (Figure 3b,c). Note that the SOM algorithm clusters genes with similar methylation profiles among the samples together into the spot-like areas appearing in the methylation maps. Accordingly, groups of signature genes with characteristic methylation profiles can be extracted from these spots assigned using Arabic numbers (Figure 3c). The methylation maps thus provide genes hyper- and hypomethylated in lymphoma compared with B-cells and also genes with almost invariant methylation levels. For example, genes in region 5 are clearly on high methylation level in B-cells and on lower level and thus hypomethylated in lymphoma.

### 3.3. Portraying of Differential Methylation Better Resolves Differences between the Lymphoma Classes

**Figure 4:**
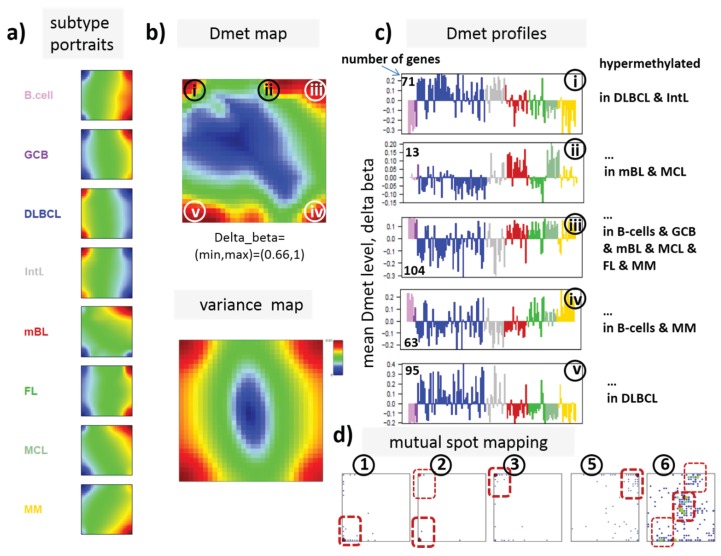
Differential methylation portraying of lymphoma and controls (DmetSOM; see legend of Figure 3 for a detailed description of the panels (**a–c**)). The DmetSOM better resolves differential methylation between the lymphoma classes (compare with the MetSOM in Figure 3). In part d genes from methylation spots 1–6 of the MetSOM are mapped into the DmetSOM (each dot marks a metagene occupied by at least one gene from each of the spots 1–6, respectively). One finds almost a 1:1 relationship between the spots except for spot 6 which “hides” spot ii. Lists of genes from the regions i–v are given in Table S2.

In our previous work, we showed that the analysis of centralized values better resolves subtle differences between the samples [22]. We therefore calculated a second SOM using centralized methylation values (DmetSOM), where the mean beta value of each gene averaged over all samples was subtracted from its actual beta-value. Centralization rather focuses the view on methylation changes between the samples independent of the absolute methylation level of the genes. In the obtained DmetSOM portraits, we identified five spot-clusters numbered i–v, which provide differential methylation profiles reflecting specific hyper- and hypomethylation of selected lymphoma classes compared with B-cells. (Figure 4a–c). Invariantly methylated genes accumulate in the center of the map, whereas the variable genes occupy different regions near the border in a profile-specific manner. Mapping of the methylation clusters 1–6 obtained from the MetSOM (previous subsection) into the DmetSOM reveals mostly a one-to-one relationship (Figure 4d). For example, spot v referring to genes specifically hypomethylated in B-cell, mBL and MCL compared to DLBCL distributes over spots 1 and, to a less degree spot 2. This result simply means that most of the genes undergoing hypo- or hypermethylation between the different sample classes show predominantly an initially high or low methylation level, respectively. We therefore restrict our further analysis to the clusters i–v in the MetSOM.

Gene set enrichment analysis provides first ideas about the functional context of the genes in the spot modules (Table 1). Spots i and v hypermethylated in DLBCL and IntL enrich genes related to the formation of the polycomb repressive complex (PRC2), which controls cellular development and differentiation [23]. Interestingly, genes from these spots are hypermethylated also in other cancers such as colorectal cancer (CRC) and high and low grade glioma. Vice versa, hypomethylated genes in DLBCL and IntL (spot iii) are also consistently hypomethylated in CRC and glioma suggesting parallels in epigenetic regulation between different cancer types. Genes hypermethylated in B-cells and MM (spot iv) are associated with immune processes, whereas genes hypermethylated in mBL and MCL (spot ii) enrich processes related to cell proliferation and cell cycle activity.

**Figure 5:**
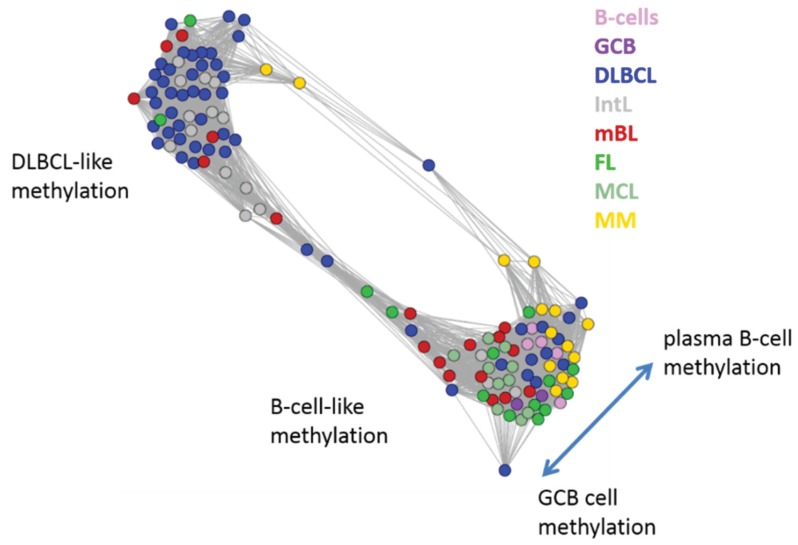
Similarity network of the methylation landscapes of the lymphoma samples studied. Each circle refers to one sample colored according to its class assignment. Samples with strong correlations between their methylation landscapes were connected by lines (r > 0.5, see [5] for methodical details). Two main cluster can be distinguished, which include samples of B-cell-like and DLBCL-like methylation.

Next, we investigate the diversity landscape of the methylation portraits of the lymphoma and reference samples. The calculated similarity network reveals two main clusters, which can be assigned to samples methylated either similarly to B-cells or to DLBCL (Figure 5). The essentially two main spot patterns of the mean DmetSOM portraits shown in Figure 4a directly reflect the separation between two main sample clusters seen in Figure 5: The samples with DLBCL-like methylation patterns preferentially show red hypermethylation spots in the left part of the portraits (spots i and v, see also Table 1), whereas the B-cell-like methylation patterns is characterized by red hypermethylation spots in the right part of the map (spots ii to iv). These patterns are strongly anti-correlated (see Figure S2), *i.e.*, hypermethylation is opposed by hypomethylation for many genes when compared with mean methylation level averaged over all samples.

**Table 1:** Functional context of the differentially methylated gene clusters.

Spots			Regulated Classes		Functional Context
Dmet-Spot	Met-Spot	Mean Met-Level	Dmet up ^1^	Dmet down ^1^	Enriched Gene Sets ^2^
**i**	2,3	intermediate	DLBCL, IntL	B-cell, GCB, FL, MM	CIMP high-vs-low hypermethylated; hypermethylated-in-CRC [24]; Suz12-, Nanog- and Eed-targets [Wang]; Hypermethylated_in-cancer-and-ageing [25]; hypermethylated in primary glioblastoma [26]
**ii**	6	low	BL, MCL	DLBCL, FL	Myc-targets [27]; GO_BP: G1/S-transition in mitotic cell cycle; GO_BP: cell cycle
**iii**	5	high	B-cell, GCB, mBL, FL, MCL, MM	DLBCL, IntL	Hypomethylated in CRC; CIMP high-vs-low hypomethylated [24]; Hypermethylated in adult brain [28]; Hypomethylated in secondary glioblastoma [26]
**iv**			B-cell, MM	DLBCL, IntL, mBL	GO_BP: immune response; hypomethylated in glioma [26]; GO_CC: nuclear chromatin; NKF-beta down in mBL [2]; IL21 targets down [29]
**v**	1,2	low, intermediate	DLBCL, IntL	B-cell, GCB, mBL, FL, MCL, MM	Suz12 targets [30]; hypermethylated in grade 3 astrocytoma and grade 2 oligodendroglioma [26]; hypermethylated in low grade glioma [31]; hypermethylated in CRC [24]; low expression TF [32]

^1^ sample classes showing high (Dmet up) or low (Dmet down) methylation levels, respectively; ^2^ enrichment of predefined gene sets in the spot-lists of genes (Dmet-spot and/or Met-spots) was calculated as described in [17]. Gene sets were taken from literature or from gene ontology (GO) categories biological process (BP) or cellular component (CC).

The DLBCL-like methylation cluster contains most of the DLBCL (69%) and IntL (81%) samples but also a certain number of FL (14%), mBL (28%) and MM (14%). On the other hand, also the second cluster of B-cell-like methylation contains 25% of the DLBCL and 19% of the IntL samples. Hence, methylation of the lymphoma classes is characterized by a certain degree of fuzziness. The gallery of individual DmetSOM portraits shown in Figure S1 indicates that, e.g., two of the FL samples show clearly a DLBCL-like methylation characteristics, whereas the majority of the FL are compatible with B cell like methylation patterns. Note also that the B-cell-like methylation cluster reveals a fine structure which separates MM and B-cells on one hand and mBL, GCB cells and MCL on the other hand. This fine structure is related to subtle methylation differences between hypermethylation spots ii–iv (Figure 4 and Table 1). Finally note that similarity analysis is based on a relatively small selection of less than 800 genes only, which might distort similarity relationships if relevant groups of genes are under- or over-represented.

### 3.4. Gene Expression Portraying

**Figure 6:**
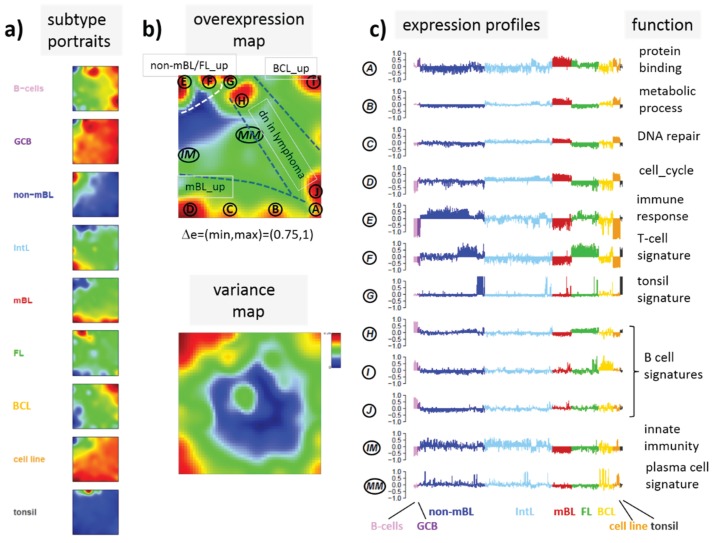
SOM portraying of the expression landscape of lymphoma (DexSOM; see caption of Figure 2). Expression classes were color-coded such that their color agrees with the respective histological class in the methylation data set.

We previously characterized the heterogeneity of gene expression landscapes of lymphoma in detail using SOM in an analogous approach as used above for differential methylation data [5,6]. Figure 6 summarizes the main results of this DexSOM analysis showing the mean SOM expression portraits of lymphoma subtypes and controls, the spot summary and variance maps and the respective spot profiles. Most of the spot modules detected can be clearly assigned to distinct lymphoma classes providing lists of signature genes which are up-regulated in the respective sample classes and which are associated with distinct biological functions. For example, mBL (spots A–D) and DLBCL (spot E) are related first of all to genes promoting proliferation and immune response, respectively.

### 3.5. Mutual Mapping of Expression and Methylation Modules

After separate SOM mapping of DNA methylation and gene expression data we linked both types of analyses in the next step to detect mutual relationships between promoter methylation and gene expression. In a first attempt, we mapped the approximately 800 genes considered on the methylation arrays into the gene expression landscape of lymphoma (DexSOM) and color-coded their methylation level (Figure S3). No densely populated areas of uniquely methylated genes were found indicating a fuzzy relationship between co-methylated and co-expressed genes. Possibly, this mutual mapping on gene level provides a suitable approach if the methylation assay probes all genes, which are also considered in the expression assay.

**Figure 7:**
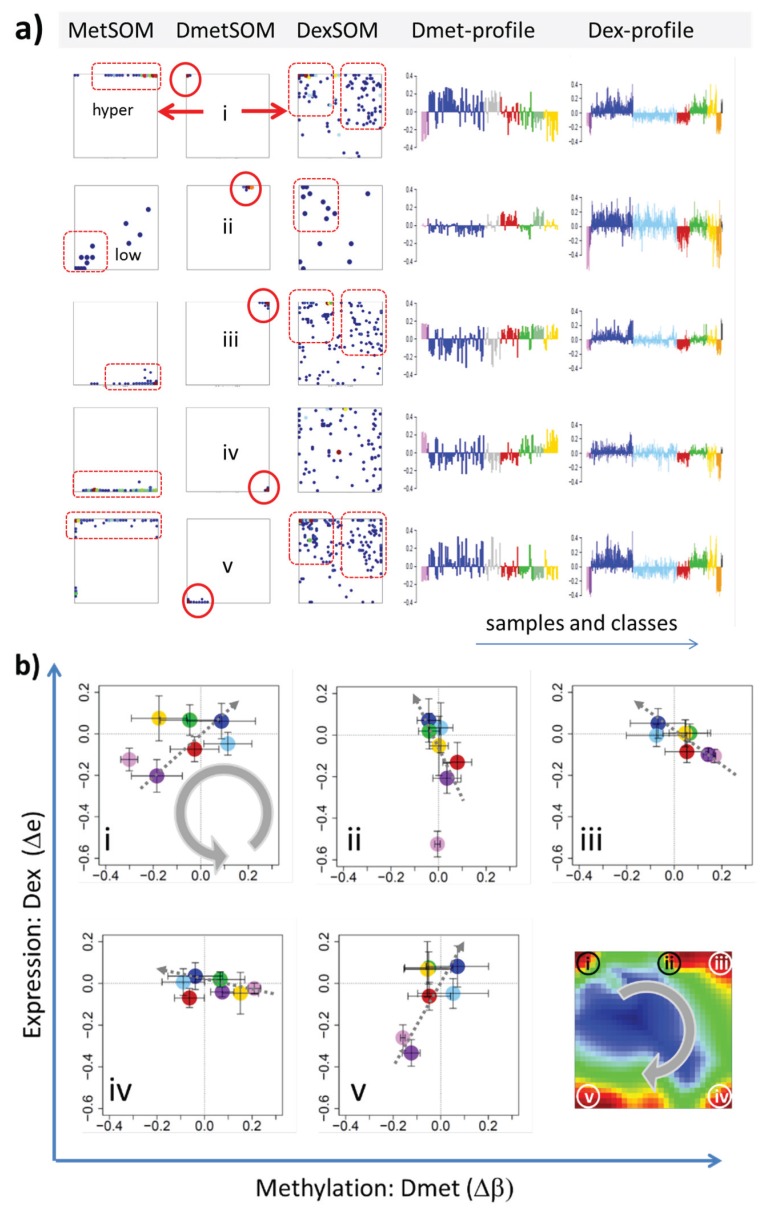
Mapping of differentially methylated genes into the expression SOM: (**a**) The Dmet-gene clusters i–v (red circles) were mapped into the Met- and Dex-SOM where they accumulate in specific areas (red rectangles). The red arrows illustrate the mapping direction; The Dmet- and Dex-profiles reveal class-specific correlations between methylation and expression data which are plotted in panel (**b**) for genes taken from each of the Dmet-spots i–v. The class-averaged mean methylation and expression levels of the gene groups were plotted in x and y directions, respectively, each class represented by a colored dot. The error bars indicate the standard deviation of the sample data of each class. The dotted arrows point from the GCB cell to the DLBCL dots thus serving as indicator for the slope of the mutual association between the methylation and expression data. Note that the clockwise arrangement of the spots i–v in the DmetSOM transforms into counter-clockwise arrangement of the data cloud in the correlation plots (see Discussion section).

In the next step, we considered groups of co-methylated genes separately: genes of the Dmet-spots i–v (see Figure 4 and red circles in Figure 7a) were mapped into the MetSOM and DexSOM where they clearly accumulate in distinct regions as indicated by the dotted red rectangles in Figure 7a. This result reflects the fact that groups of co-methylated genes are also co-expressed in a class-specific fashion as confirmed also by the respective methylation and expression profiles shown in the right part of Figure 7a. To better resolve the mutual relationships, we correlate class-averaged mean methylation and expression levels of the gene groups taken from each of the methylation spots i–v in panel b of Figure 7. Spots i and v are characterized by a positive correlation: *i.e.*, hypermethylation in DLBCL with respect to B- and GCB cells is accompanied by overexpression in DLBCL with respect to the healthy cell controls. MM and partly FL show concerted coexpression with DLBCL but still similar methylation compared with B and GCB cells. The other lymphoma classes behave similarly however with smaller effects. Genes from spot iii show a negative correlation where differential methylation changes sign compared with spots i and v but differential expression does not. In other words, overexpression in DLBCL is associated with hyper- (spots i and v) and hypo- (spot iii) methylation as well. Recall that all three spots i, iii and v are also functionally related: They enrich genes differentially methylated in other cancer types and related to PRC2 formation. Spot ii (related to proliferation, see Table 1) collects genes weakly responding to methylation but strongly to differential expression for most of the lymphoma classes. Note that in mBL hypermethylation of spot ii genes associates with underexpression of the respective genes. In contrast, spot iv (related to immune response) weakly responds to expression changes but strongly to differential methylation.

In Figure S5 we mapped genes differentially methylated in the histological lymphoma classes with respect to B-cells as determined in ref. [15] into our SOMs. Most of the genes hyper- or hypomethylated in lymphoma accumulate in spot i or iii (and iv), respectively, showing similar correlations with expression data as discussed above. We also studied genes specifically hypermethylated in classical Hodgkin lymphoma compared with B cells determined in ref. [8] (Figure S6). Interestingly these genes accumulate in expression spots A, H and MM which mostly associate with healthy GCB-cell functions but lack differential methylation in our data presumably because of the absence of Hodgkin lymphoma cases in our data.

We also mapped the spot-clusters of co-expressed genes extracted from the expression SOM into the methylation SOM to assess mutual correlations (see supplementary Figure S4). Most of the effects observed are weaker than for the co-methylated gene clusters i–v presumably due to causal relationships between promoter methylation and gene expression leading to the dilution of correlations in the opposite direction. On the other hand, the data clearly reveal expression changes between lymphoma and the reference B and GCB cells, which are accompanied by marked differential methylation effects in both positive and negative directions as well.

Hence, we observe positive and negative correlations between expression and methylation changes by mapping clusters of co-methylated genes into expression space and vice versa. The most pronounced effects are observed between B/GCB cells and DLBCL in correspondence with the sample diversity analysis (Figure 5), but also the other lymphoma subtypes show gradual and specific effects roughly in the same order as illustrated in Figure 2c.

### 3.6. Mapping of Functional Gene Sets Indicates a Distinguished Role of PRC2 Genes

**Figure 8:**
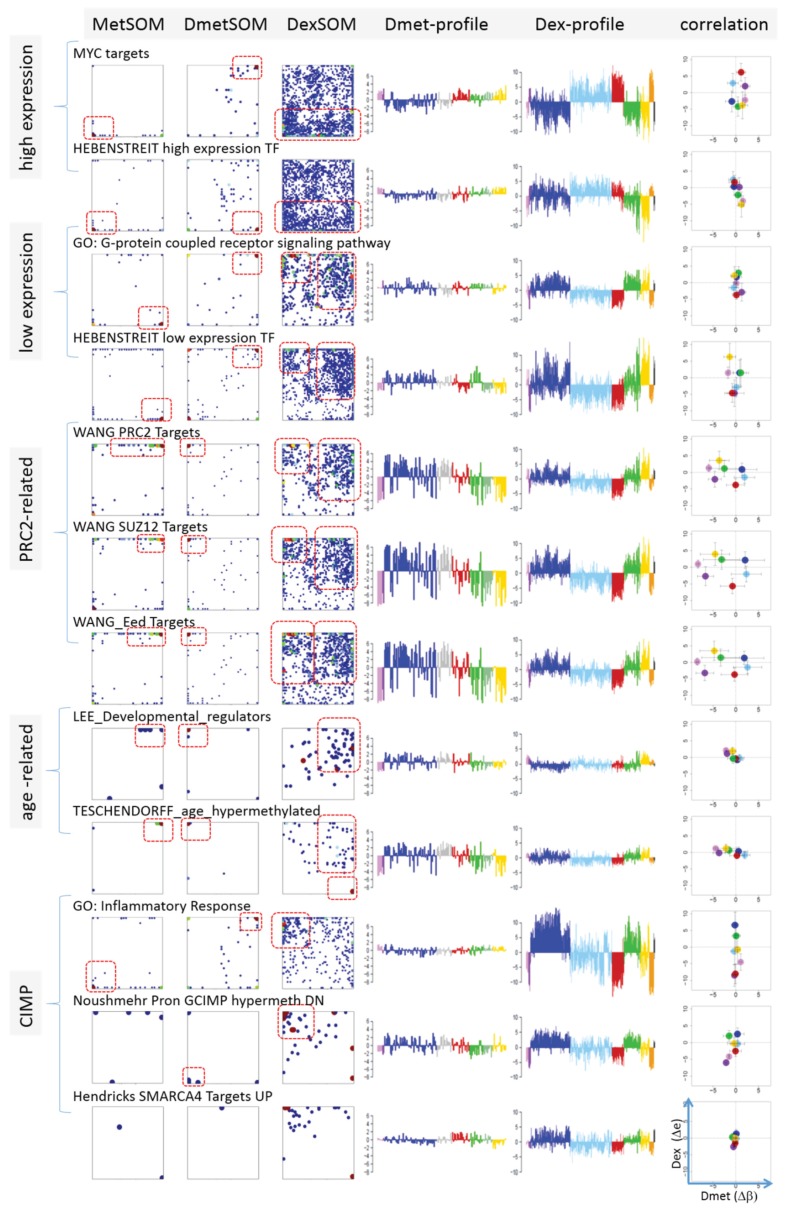
Mapping of selected functional gene sets into the methylation and expression SOM (see legend of Figure 7 for assignments). Gene sets were taken from [30,32,33,34,35,36,37,38]. The combined methylation-expression data group into five different patterns as indicated by the brackets and the designations given at the left part of the figure. An enlarged version of the correlation plots for selected gene sets is given in Figure S7.

Next, we analyzed a series of functional gene sets in an analogous fashion as the spot modules in the previous subsection (Figure 8). The obtained characteristics can be grouped into different patterns. *MYC* targets [33] and transcription factors (TF) associated with high gene expression levels [32] give rise to large expression differences between the lymphoma classes but almost negligible methylation effects. TFs associated with low expression levels and G-protein receptors show a similar relationship between expression and methylation changes where the expression levels of the lymphoma classes however swap their order in the correlation plot. The latter effect can be directly extracted from the areas of highest population densities of the genes in the DexSOM: The “high expression” genes enrich the lower part of the DexSOM whereas the “low expression genes” preferentially occupy areas near the left and right upper corners of the map (see the dotted red rectangles in Figure 8). The third group of PRC2-related genes gives rise to marked class-specific expression and methylation changes. Interestingly, the expression characteristics of the PRC2-group and of the “low expression” group are almost identical whereas their methylation characteristics differ largely in amplitude. It seems that the PRC2-related gene sets specifically select genes which change expression and methylation in a lymphoma-specific fashion whereas the low expression gene sets contain genes which show main effects in the expression domain only. This difference can be rationalized by the fact that a large fraction of these genes is affected “indirectly” by downstream co-regulation of gene expression without alterations of promoter methylation.

The next group of “age related genes” can be interpreted as a subgroup of the PRC2-related and low expression genes which occupies essentially only the right upper region of the DexSOM. In the correlation plot, one sees that this restriction strongly reduces the variance of the expression values between the lymphoma classes whereas the alterations of methylation are similar to the PRC2-related gene sets. This result implies that PRC2-related genes are governed by more diverse regulation mechanisms of gene expression than the age-related genes. Note however that the gene set “developmental regulators” being part of the “age-related” group also collects genes referring to the formation of the polycomb complex [34]. These genes were obtained from gene expression measurements whereas the “ageing-associated hypermethylated genes” [35] are extracted from DNA methylation studies which explains the larger response of the latter ones in the methylation dimension.

The last “CIMP”-group genes accumulate in the top left region of the DexSOM. They consequently share similarities with the groups of “PCR2-related” and “low expression genes” whose genes also accumulate in this region of the map. The methylation effect of the gene sets’ “inflammatory response” is small but more pronounced for the GCIMP-gene set extracted from glioma data [36]. Other CIMP- and GCIMP-related gene sets obtained in colorectal and in brain cancer studies, respectively, also respond in the methylation dimension (see Table 1).

In summary, we found two main combined methylation/expression patterns exemplified by the “high-expression” and “PCR2-related” groups where only the latter is characterized by both expression and methylation changes. The latter group can be further split into CIMP-like and “age” related genes.

### 3.7. EZH2 Targets Strongly Deregulate in Lymphoma

*EZH2* is the catalytic subunit of the PRC2 and mediates transcriptional repression through its histone methyltransferase activity that trimethylates H3K27 [39,40]. *EZH2* is upregulated in normal GCB cells and it is implicated in lymphomagenesis. Its targets overlapped extensively between GCB cells and embryonic stem cells [39] and are preferentially trimethylated lysine 27 on histone H3- (H3K27me3, about 70%) and thus transcriptional inactive. *EZH2* in normal GCB cells favors cellular proliferation by directly repressing several tumor suppressor genes, creates a repression state similar to that found in stem cells that might foster self-renewal and prevent premature differentiation, and maintains and stabilizes a transcriptional repression program already in place in naïve B-cells [37].

We analyzed *EZH2* targets and H3K27me3-marked genes determined in hESC, (naïve) B and GCB (centroblasts) cells obtained by means of ChIP-chip experiments [39]. The respective sets of genes were mapped into our different SOMs (Figure S8). The methylation and expression characteristics of both *EZH2* target and H3K27me3-marked genes in hESC, B and GCB cells among the systems studied here are very similar and closely resemble those of the PRC2-related genes (compare Figure S8 with Figure 8). These genes show both low expression and methylation in mBL, B and GCB cells and increased expression in DLBCL, IntL, FL and MM. This result suggests that transcription is repressed in H3K27me3-marked *EZH2* targets in healthy B and GCB cells, and that repression of these targets is mostly maintained in mBL but at least partly turned into activation in DLBCL, IntL, FL and MM. These expression changes are paralleled by hypomethylation in B and GCB cells and in MM on one hand and hypermethylation in DLBCL and IntL on the other hand. Stratification of genes with respect to anti-correlation between expression of *EZH2* and that of its targets [39] specifically selects genes from DexSOM spots E and F supporting this view because *EZH2*-mediated trimethylation of H3K27 is expected to inactivate the expression of the *EZH2* target genes. Interestingly, *de-novo EZH2* targets in centroblasts compared with naïve B-cells (NB) reverses expression levels and to a less degree also methylation levels in MM, B and GCB cells. It was suggested that *EZH2* upregulation during the transition from NB cells to centroblasts reactivates a stem cell-like repression program, which is not present in NB cells and possibly featuring increased self-renewal and proliferative potential [39].

Velichutina *et al.* [39] also compared absolute expression levels between *EZH2* targets and non-targets serving as reference. We found similar results, particularly a reduced mean gene expression of *EZH2* targets in all sample classes studied if one considers all *EZH2* targets identified in B-cells (Figure S9). The difference of expression levels between targets and non-targets however largely reduces if one considers only *EZH2* targets showing negative correlation of their expression levels with expression of the *EZH2* gene. This difference even changes sign for targets showing positive correlations with the *EZH2* gene expression. This result shows that total gene expression markedly varies between different groups of genes where the origin of this effect is not clear. The total gene expression level of *EZH2* targets changing in concert with *EZH2* is the largest, for anticorrelated targets it is intermediate, and for targets with low correlation it is at the lowest level. Transcriptome-wide MYC hyperactivation of the first group, and chromatin reorganization and promoter methylation of the second and third groups, are potential and mutually overlapping factors that modulate total expression (see below).

### 3.8. Chromatin States and Their Remodeling

Higher-order chromatin structure is emerging as an important regulator of gene expression. Alterations of gene expression programs can be induced by the remodeling of chromatin states which, for example, facilitate transcription in open regions of euchromatin, but prevent gene expression in dense packed regions of heterochromatin. These different states of chromatin conformation are governed by the arrangement of nucleosomes being the central structural elements of DNA packing in the nucleus. In turn, the arrangement of nucleosomes is modulated by chemical modifications of distinct amino acids in the side chains of the histone units forming the nucleosomes. A whole battery of such modifications and their combinatorial patterns are able to tune the transcriptional activity of the affected genes by influencing the functional state of gene’s structural elements such as enhancers and promoters, and also stages of the transcriptional process such as transcriptional elongation, transition, activation and repression [41].

To get insight into the possible mechanism of chromatin remodeling in lymphoma, we make use of the chromatin states identified in GM12878 lymphoblastoid cells (LBC) which imitate immature lymphocytes [38]. The chromatin states were calculated from ChIP-Seq data of a series of histone modifications using a hidden Markov model [42]. We mapped the respective chromatin regions of each state on the human genome and collected the genes included in each of the 11 chromatin states into one gene set, and then mapped them into the lymphoma methylation and expression SOMs to assess their methylation and expression characteristics as described above (Figure S10).

We found close correspondence between the methylation/expression properties of groups of chromatin states and the groups of functional gene sets identified above: Genes with chromatin states strongly promoting transcription (active Txn-states), namely the states “active promoters”, “transcriptional elongation” and “transcriptional transition”, “weak transcription” and also the state “weak promoter” closely resemble the characteristics of the “high expression” gene sets shown in Figure 8. Contrarily, transcriptionally inactive states (“poised promoters” and “repressed promoters”) share close similarity with the PCR2-related gene sets. The state “heterochromatin” resembles the “low expression” gene sets. Note that the Txn-inactive states and “heterochromatin” show a nearly mirror symmetrical profile of gene expression compared with the Txn-active states with low expression levels in mBL and IntL and high levels in BCL (MM), non-mBL and FL. The state “strong enhancers” forms a separate group which differs from the functional gene sets considered. Its methylation and expression profiles virtually agree with that of the active Txn-states except for the expression level in mBL which turns from high activity in the latter states into low activity in the former one and vice versa for FL.

Interestingly, the transcriptional inactive chromatin states (and also PRC2-related genes) show the largest variability of DNA methylation between the classes with lowest levels in healthy GCB and B-cells, intermediate levels in MM, FL and mBL, and high levels in DLBCL and IntL. Thus, they resemble the order of overall methylation variability shown in Figure 2. These methylation changes were paralleled by positively correlated alterations of gene expression. Genes located in heterochromatin show virtually the same class-dependence of gene expression but almost no variation in methylation. Hence, genes becoming activated in heterochromatin are obviously affected by other mechanisms not associated with methylation changes of their promoters.

**Figure 9:**
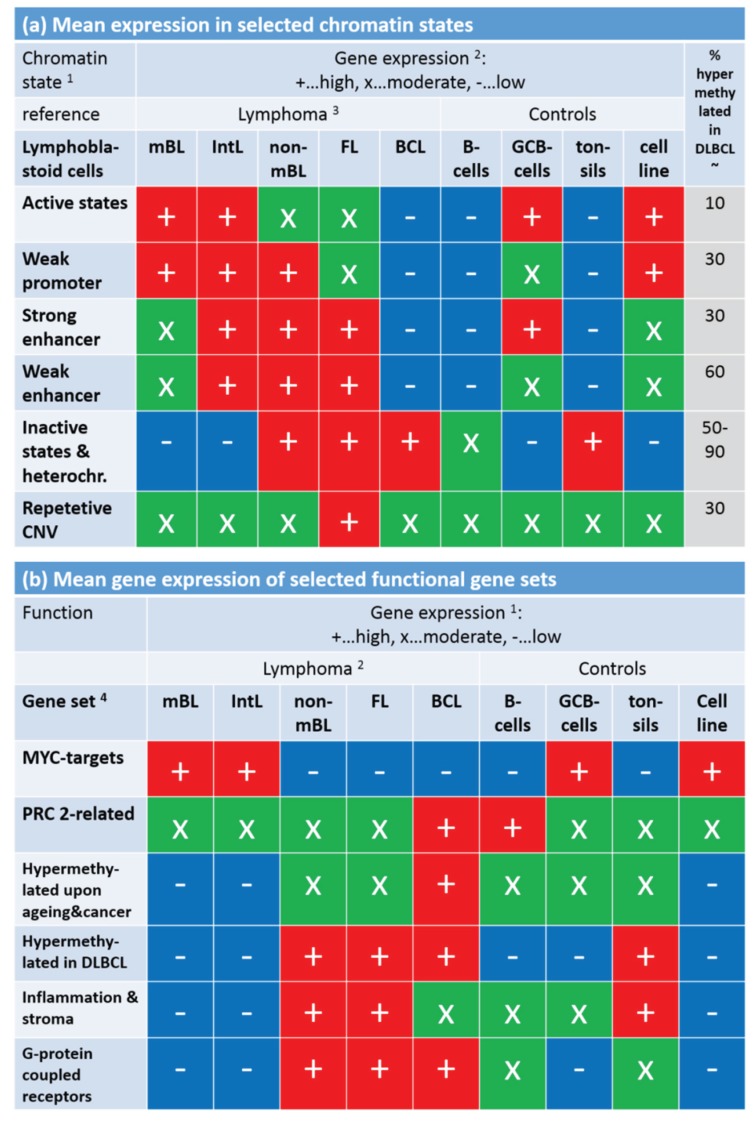
Mean gene expression level of selected chromatin states in the lymphoma and reference systems (part **a**) and of selected gene sets (part **b**). Gene expression was stratified into high (red), moderate (green) and low (blue) levels using the respective GSZ-profiles. ^1^ Chromatin states were defined in [42] with respect to the associated histone marks (see, e.g., Figure 1b in [42]). The most characteristic marks are in active states (e.g., active promoters): H3K4me3/me2, H3K27ac, H3K9ac; weak promoter: H3K4me3/me2; strong enhancer: H3K4me1/me2, H3K27ac, H3K9ac; weak enhancer: H3K4me1/me2; inactive states (e.g., inactive and poised promoters): H3K27me3, H3K4me2; heterochromatin: no mark; repetitive CNV: all marks; ^2^ gene expression levels of the chromatin states (Figure S10) and functional gene sets (Figure 8); ^3^ assignment of lymphoma classes refers to the expression classes introduced in Figure 6; ^4^ Gene sets were taken from [33] (MYC), [34] (PRC developmental regulators), [35] (Hypermethylated upon ageing and cancer), [15] (hypermethylated in DLBCL, [3,43] (inflammation and stroma) and GO (G-protein coupled receptor activity and signaling pathway); see also Figure 8.

Note that the assignment of chromatin states refers to the lymphoblastoid cell line but not to the lymphoma classes studied here. Generally, one expects that Txn-active states show higher gene expression levels than Txn-inactive states and heterochromatin. This trivial relationship implies using the mean transcriptional activity of the chromatin states in lymphoma as a measure to estimate the correspondence between the nominal chromatin state referring to lymphoblastoid cells and the real one in lymphoma. For an overview, we stratified the expression levels of the chromatin states in the different lymphoma classes into high, moderate and low levels based on the GSZ-profiles shown in Figure S10 and visualized them in Figure 9a: The expression level observed in GCB cells, mBL and IntL is indeed high in Txn-active chromatin states and low in Txn-inactive chromatin states. Thus, the real expression levels agree with the nominal ones suggesting global correspondence between the chromatin states in the reference cells and that in mBL and IntL. Contrarily, the expression levels in BCL, FL and non-mBL disagree with the expression levels expected for the nominal chromatin states: High expression levels in the lymphoma subtypes were observed for genes assigned to inactive states in the reference cells and reduced expression levels were observed for genes in states of high transcriptional activity of the lymphoblastoid cells. Hence, the expression levels observed in mBL and IntL, in GCB cells and the cancer cell line are in correspondence with the chromatin states in lymphoblastoid cells, whereas the expression levels observed in B-cells, BCL, FL, non-mBL and also tonsils contradict them. This switching of gene activity between these two groups of samples suggests remodeling of chromatin in BCL, FL and also non-mBL compared with lymphoblastoid cells and thus also with mBL, IntL and GCB cells. Note also that the activity patterns of B-cells, tonsils and also of BCL differs from that of GCB cells suggesting remodeling of chromatin between healthy (pre- and post-GC) B-cells and GCB cells. Moreover, the similar expression patterns of B-cells and of BCL supports the plasma cell characteristics of BCL differing from the characteristics of the GC-derived lymphoma subtypes.

To assess the relationship between DNA hypermethylation in lymphoma and the chromatin states, we calculated the percentage of overlap-genes from the different chromatin states also found in the set “hypermethylated in DLBCL” taken from [15]. The overlap of hypermethylated genes is only about 10% for transcriptional active states but much higher (50%–90%) for transcriptional inactive states. Hence, activation of the latter states in DLBCL/non-mBL and FL seems to be accompanied by hyper-methylation of a large fraction of genes being inactive in lymphoblastoid cells, mBL and IntL.

Finally, we transferred the expression levels of selected gene sets discussed above into the tabular form for direct comparison with that of the chromatin states (Figure 9b). *MYC* target genes are expressed in parallel with Txn-active states among the systems studied. This agreement suggests that the *MYC* targets are found predominantly in chromatin regions active in mBL, IntL, GCB cells, the cancer cell line and lymphoblastoid cells (95% overlap between *MYC* targets and active promoters). In contrast, gene sets related to inflammation and G-protein receptor activity both hypermethylated in DLBCL accumulate in chromatin states inactive in the reference system but activated in DLBCL, IntL, FL and also BCL.

In summary, gene sets referring to distinct chromatin states in the reference cells show well distinguished expression and DNA methylation characteristics either agreeing or disagreeing with the expression level expected in the nominal chromatin states. Disagreement indicates chromatin remodeling in IntL and non-mBL and especially in B-cells and BCL compared with mBL and GCB cells. Hence, one can distinguish three groups of samples showing characteristic expression patterns of genes assigned to different chromatin states. They comprise (i) mBL, GCB cells and cancer cell lines; (ii) BCL, MM and (pre- and post-GC) B cells and (iii) IntL, DLBCL and FL.

## 4. Discussion

### 4.1. Integrative SOM Portraying Resolves Differently Methylated and Expressed Genes and Their Functional Context

Epigenetics challenges bioinformatics as it requires integration of data of different omics realms to resolve the interplay between regulatory mechanisms on genomic, epigenomic, methylomic, transcriptional and translational levels. Our study focused on gene expression and DNA methylation data stratified with respect to different histological and molecular subtypes of lymphoma and healthy controls to discover epigenetic mechanisms of tumor genesis and progression. We applied SOM machine learning to the data, a powerful technique to “organize” complex, multivariate data. Using centralized methylation and expression data we identified clusters of co-methylated and of co-expressed genes among the samples studied, which we call “spot-modules” because of their spot-like appearance in the SOM-portraits.

**Figure 10:**
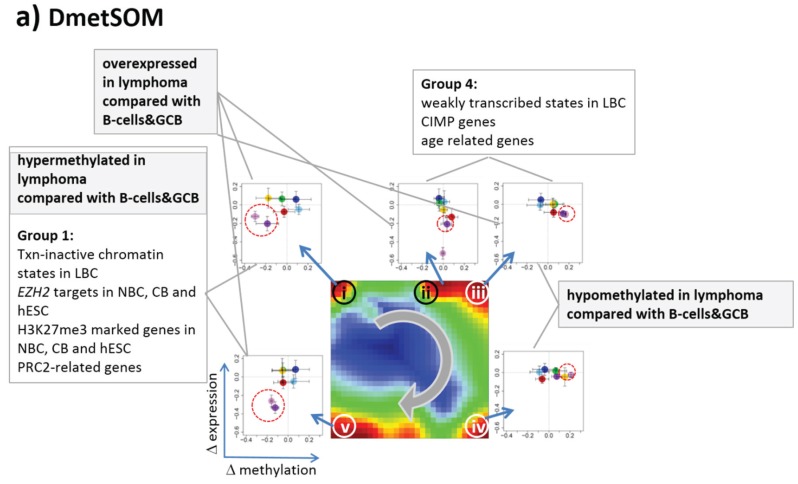

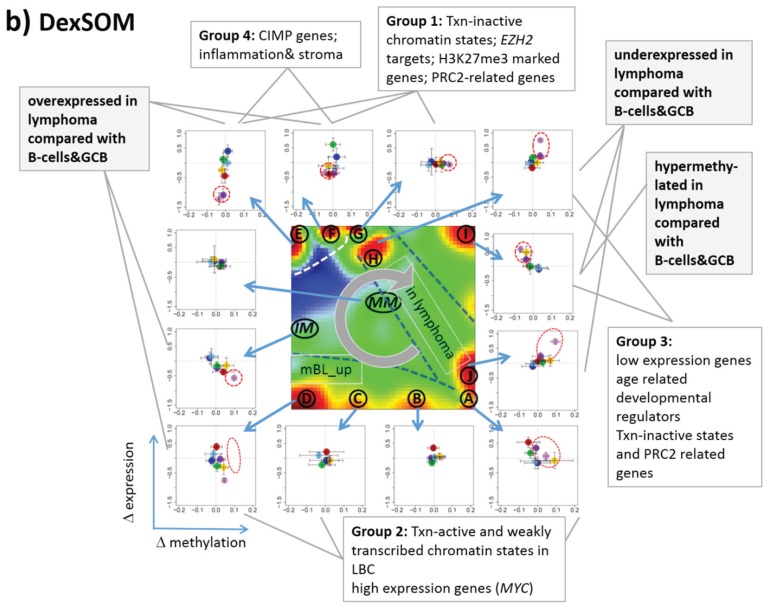
Integrative view on differential methylation and gene expression in lymphoma and on the related functional context. Spot modules of co-methylated genes were extracted from the DmetSOM (part **a**) and spot modules of co-expressed genes from the DexSOM (**b**). The class-specific correlation plots for each spot reveal systematic methylation and expression changes in both maps many of them being associated with functional gene sets. Especially, differential methylation and expression with respect to healthy controls (B-cells and GCB cells, see red dotted circles) as well as systematic differences between lymphoma subtypes (e.g., mBL, DLBCL and MM) were sorted in a systematic fashion in both SOM maps.

Mutual correlation plots between the mean expression and methylation levels of the genes of each of the spot-modules revealed different patterns with impact for underlying epigenetic mechanisms of genomic regulation (Figure 10). We identified groups of genes mostly affected by methylation with only tiny expression changes (e.g., DmetSOM-spots iii and iv and DexSOM-spot G), vice versa, groups of genes with almost invariant methylation levels but strongly varied expression (e.g., DmetSOM-spots ii and DexSOM-spots D and E), and groups with strong positive (spots i, v and H and J) and negative (e.g., spots iii, A and I) correlations between expression and methylation levels in the different sample classes. Moreover, the Dmet- and DexSOM disentangle genes systematically hyper- and hypomethylated and/or over- and underexpressed in lymphoma compared with healthy B and GCB cells (see Figure 10). Hence, SOM portraying served as an effective sorting machine to extract different modes of co-regulation between expression and methylation mechanisms specifically characterizing lymphoma and differentiating also between the lymphoma subtypes.

To assign the functional meaning to the spot modules, especially in the context of underlying epigenetic mechanisms, we applied enrichment analysis using a multitude of pre-defined gene sets related to categories such as biological function (e.g., inflammation, cell development and ageing), targets of different transcription factors (e.g., *MYC*, high and low expression TFs) and epigenetic modulators (e.g., *EZH2*, *SUZ12*; *PRC2*), different chromatin states in reference lymphoblastoid cells and also genes differently expressed and methylated in other cancers (e.g., CIMP and GCIMP genes in colorectal cancer and glioma, respectively). Interestingly, we found pronounced similarities of the expression and methylation signatures of gene sets from different categories in the lymphoma data which indicate there are mutual relationships between them. Particularly, the spot-modules can be sorted roughly into four main groups (see Figure 10):

- Group 1 is enriched in PRC2 and *EZH2* targets, related to transcriptionally inactive states in LBC and shows strong variation in expression and methylation levels being hypermethylated and overexpressed in lymphoma compared with the controls;
- Group 2 comprises transcriptionally active chromatin states, TFs related to highly expressed genes and *MYC* targets. It promotes cell proliferation and shows strong expression changes especially between mBL on high and the controls on low levels, but virtually no differential methylation;
- Group 3 accumulates mostly in the top right part of DexSOM and contains ageing and developmental genes, and low expression TF genes. It overlaps with group 1 with respect to the enriched chromatin states and part of the *PRC2* and *EZH2* targets. Expression of these genes is down regulated in lymphoma compared with the controls but the methylation can differ in both directions;
- Group 4 accumulates in the top left part of the DexSOM and contains CIMP/GCIMP genes, genes related to inflammation and stroma, *SMARCA4* targets and another part of the *PRC2* and *EZH2* targets. These genes are strongly upregulated in DLBCL, IntL and partly FL, and downregulated in the controls and BL. They show moderate methylation changes being slightly hypermethylated in lymphoma.

### 4.2. Dynamic Regulation of Epigenetic Landscapes in Lymphoma and during B-Cell Development

**Figure 11:**
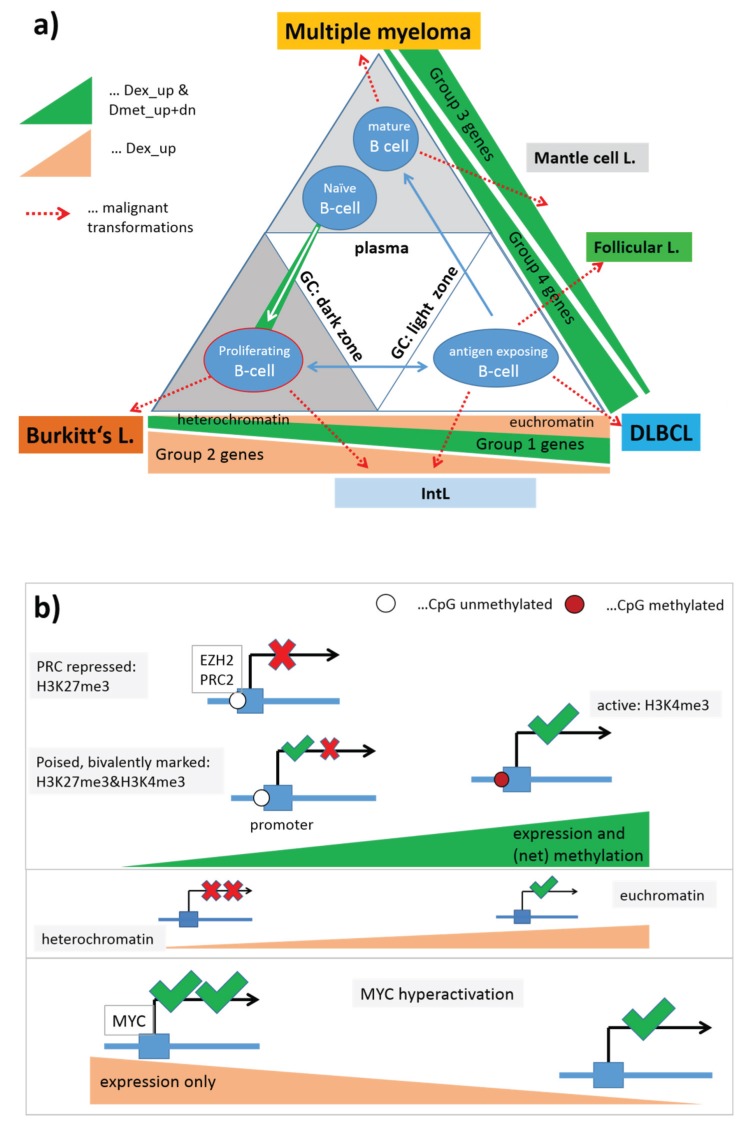
Scheme illustrating lymphoma heterogeneity with respect to their cell of origin and groups of affected genes (part (**a**)). Different lymphoma subtypes can originate from GCB cells located in the dark zone of the germinal centre (centroblasts), from its light zone (centrocytes) or from maturated plasma B cells as indicated by the dotted red arrows; Part (**b**) illustrates the associated chromatin states and their remodeling due to altering histone modifications affecting transcription. The green ramp codes increasing expression and methylation associated with chromatin remodeling from inactive and poised to active states. The light red ramp codes increasing expression without methylation changes either due to chromatin remodeling from hetero- to euchromatin or due to *MYC* hyperactivation. Gene groups are specified in Figure 10.

Figure 11 schematically illustrates and summarizes our results in the light of B-cell and lymphoma biology. Healthy B-cells pass essentially three relevant compartments, the dark and light zone of the GC and “outside-of-the-GC” which subsumes plasma, lymph node and also bone marrow (see also Figure 1). The associated types of B-cells can transform into the different lymphoma classes as illustrated by the red arrows in Figure 11a. The triangular shape of the scheme is motivated by the three different types of lymphoma classes which point to similarities with GC dark zone (DZ) B-cells in terms of proliferative activity, GC light zone (LZ) B-cells in terms of inflammatory signatures, and pre- and post-GCB cells in terms of (healthy) B-cell signatures (see also [5,6]).

The colored “ramps” code for alterations in gene expression and/or methylation between the lymphoma classes which associate with the groups of genes defined in the previous subsection and which were specified with respect to changing chromatin states (Figure 11b). Group 1 genes give rise to increasing differential expression and methylation between lymphoma and healthy B-cells with the largest effect in DLBCL. We suggest that the strong alterations in gene expression manifest chromatin remodeling from PRC-repressed and poised chromatin states into active ones associated with hypermethylation in lymphoma. Hence, group 1 genes are obviously of central importance for a mechanism of lymphomagenesis transforming healthy GCB cells into malignant ones. Recall that the largest differential effect of these genes in gene expression and methylation is observed for DLBCL. Along the axis linking mBL and DLBCL the expression changes are counterbalanced by group 2 genes which strongly upregulate in mBL compared with DLBCL almost without methylation changes. Presumably this trend is mainly caused by the activation of *MYC* in mBL (and also selected *MYC*-positive IntL cases) which, in turn, amplifies the expression of already transcribed genes giving rise to a sort of hyperactivation of the transcriptional state without strong DNA-methylation effects and chromatin remodeling. Group 3 and 4 genes mainly differentiate between DLBCL and MM, however, in opposite directions. Both groups show alterations in gene expression and methylation as well, and thus partly resembling group 1 genes in their molecular determinants. Particularly, group 1, 3 and 4 genes contain *PRC2* and *EZH2* targets showing that repressed and poised promoter states play a pivotal role in cell fate decisions of GCB cells and in their transformation into cancerogenic states.

B-cells employ epigenetic mechanisms to generate effective memory responses resembling epigenetic reprogramming of stem cells upon cell fate decisions. Particularly, the transition from naïve B-cells permits GCB cells to generate the differential response to antigenic challenges and to differentiate toward plasma cell fates. Deregulation of the underlying epigenetic determinants such as DNA methylation [7] and/or chromatin activity states can be assumed to potentially disturb or even to prevent normal differentiation of B-cells leading to malignant lymphomas.

Note that not only the transition between naïve B and GCB cells requires alterations of the cellular programs but also the polarization of transcriptional programs between LZ and DZ in normal GCB cells suggests that environmental cues encountered by B-cells when moving between GC compartments determine their phenotype [44,45,46]. Normal LZ and DZ GCB cells represent alternating states of the same cell population. Hence, the transition between naïve B-cells and GCB cells and between DZ and LZ and vice versa requires a certain degree of plasticity of the underlying transcriptional programs to switch between the underlying more proliferative centroblastic and the more inflammatory centrocytic B-cell phenotypes.

Many promoters in embryonic stem cells harbor a distinctive histone modification signature that combines the activating histone H3 lysine 4 trimethylation (H3K4me3) mark and the repressive H3K27me3 mark. These bivalent domains are considered to poise expression of developmental genes, allowing timely activation while remaining repressed in the absence of differentiation signals [47]. Hence, bivalent domains and associated chromatin-modifying complexes safeguard proper and robust differentiation. In view of this basal mechanism, it appears not surprisingly that bivalent chromatin states in the reference lymphoblastoid cells are strongly affected by expression and methylation changes observed in group 1, 3 and 4 genes. These bivalent promoters possibly ensure the plasticity of the genome to switch between the functional requirements in the different compartments of the GC. Moreover, it was previously shown that genes *de novo* methylated in lymphoma subtypes are enriched for *PCR2* targets in embryonic stem cells showing that lymphoma share a similar stem cell-like epigenetic pattern either because lymphoma originate from cells with stem cell features or because stemness is acquired during lymphomagenesis by epigenetic remodeling [8].

Recent studies suggest that *EZH2* upregulation during the transition of naïve B-cell to proliferating GCB cell (centroblast) reactivates this stem cell-like repression program not present in naïve B-cells and possibly featuring increased self-renewal and proliferative potential. This program accomplishes a proliferative function in GCB cells which makes them prone for malignant transformation into lymphoma [39]. Moreover, *PCR2*-mediated repression seems to be almost independent of DNA methylation in normal B-cells (including proliferating centroblasts). However, in lymphoma, DNA methylation of these genes clearly changes, where many hypermethylated genes are targeted by *PCR2* also found in stem cells [8] and centroblasts [39]. The authors of the latter paper hypothesize that lymphoma cells may have achieved a selective advantage by recruiting DNA methyltransferases to *PCR2*-bound or/and H3K27me3-marked promoters and that increased promoter DNA methylation may consolidate and stabilize *PCR2*-mediated repression of one or several of the tumor suppressors targeted by *PCR2* or make them less capable of responding to anti-oncogenic signals [37]. However, methylation in the promoters of PCR2 genes can also associate with the opposite effect by destabilizing inactive chromatin states and thus promoting their remodeling into active ones, e.g., in group 3 genes in DLBCL. Our analysis suggests also the parallel remodeling of heterochromatin into transcriptionally active euchromatin without clear alterations of the methylation of the promoters of the involved genes.

## 5. Conclusions

From a methodical viewpoint our study shows, that combined SOM portraying of expression and methylation data together with function mining using a battery of gene sets provides detailed insights into the regulatory landscape affecting the transcriptome and methylome and delivers a hypothesis for epigenetic mechanisms of lymphomagenesis. Our analysis is based on unmatched data sets with respect to the cancer cases used. We expect considerable improvement of the method for matched data sets.

Our study confirms previous results about the role of stemness genes during development and maturation of B-cells and the dysfunction of these regulatory programs in lymphoma presumably locking them in more proliferative or more immune-reactive states referring to GCB cell functionalities in the dark and light zone of the GC. These dysfunctions are governed by epigenetic effects altering the promoter methylation of the involved genes, their activity status as moderated by histone modifications and also by higher-order chromatin structures which emerge as an important regulator of gene expression.

## Supplementary Files

Supplementary File 1

Supplementary File 1

Supplementary File 1
