# Supplementary material for: Epigenetic Heterogeneity of B-Cell Lymphoma: DNA Methylation, Gene Expression and Chromatin States
Source: Genes (Basel). 2015 Sep 7;6(3):812–40. doi: 10.3390/genes6030812 (PMC4584331; doi:10.3390/genes6030812)
Supplement: Supplementary File 1 [file genes-06-00812-s001.docx]

Supplemental Materials


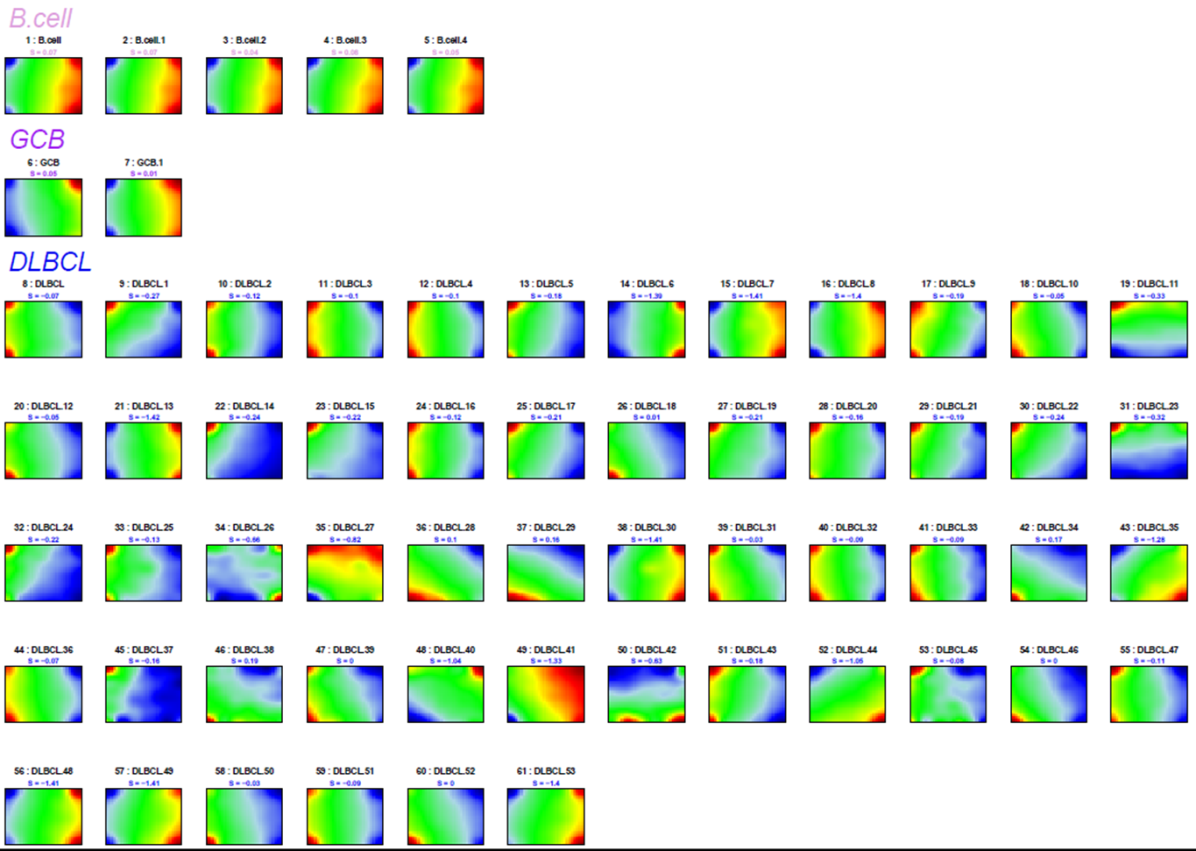


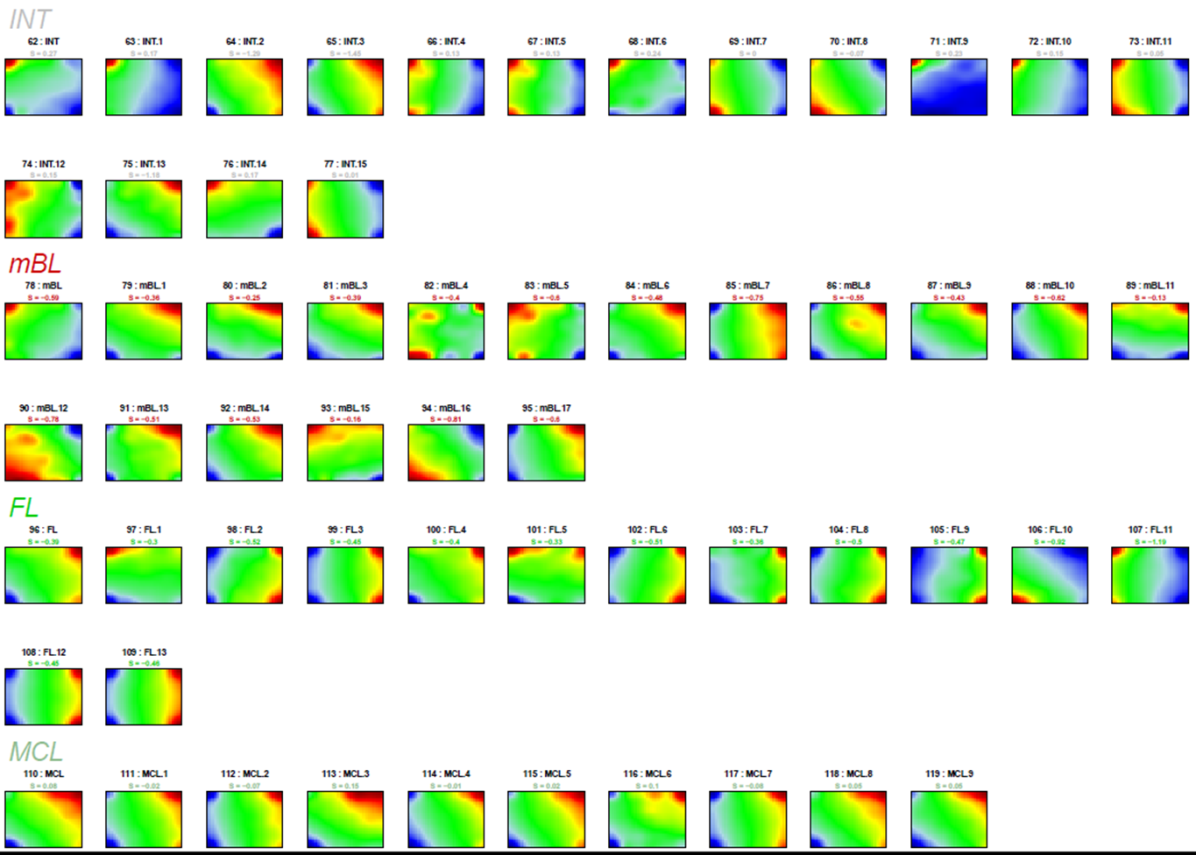


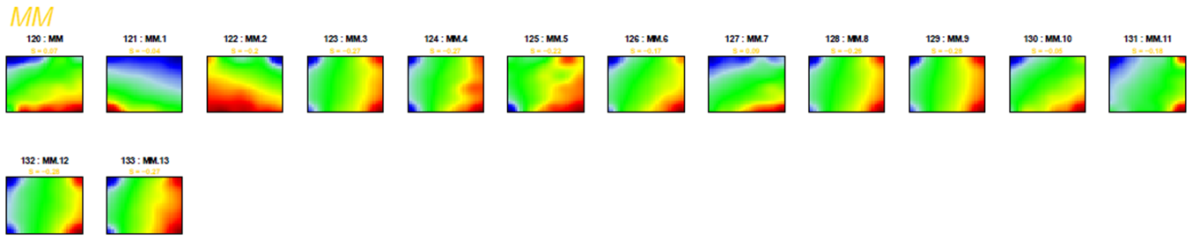


**Figure S1.** Gallery of DmetSOM portraits of all samples studied.


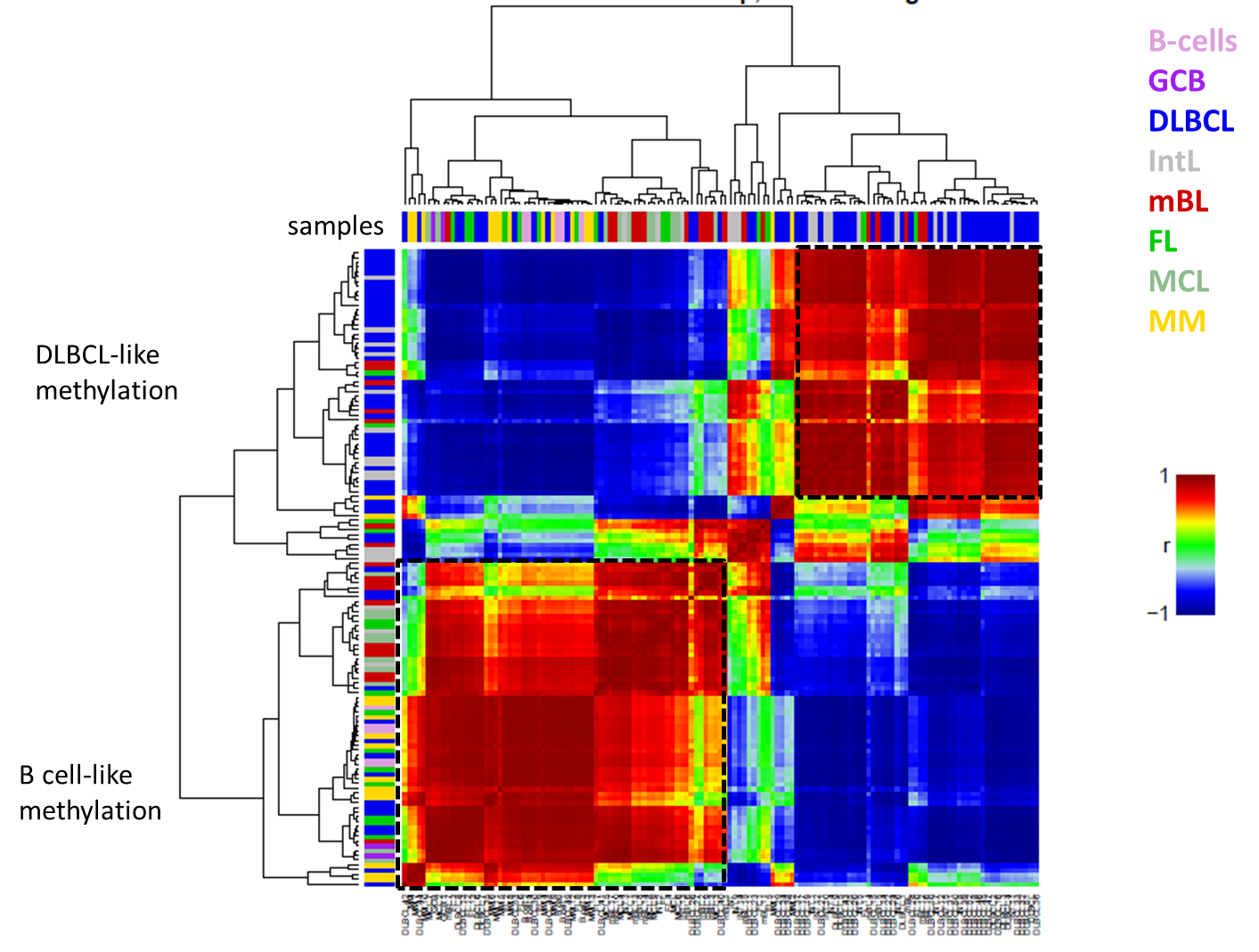


**Figure S2.** The pairwise correlation map of the DmetSOM portraits of all samples reveals strong anticorrelation between the methylation landscapes of DLBCL-like and B cell-like clusters.


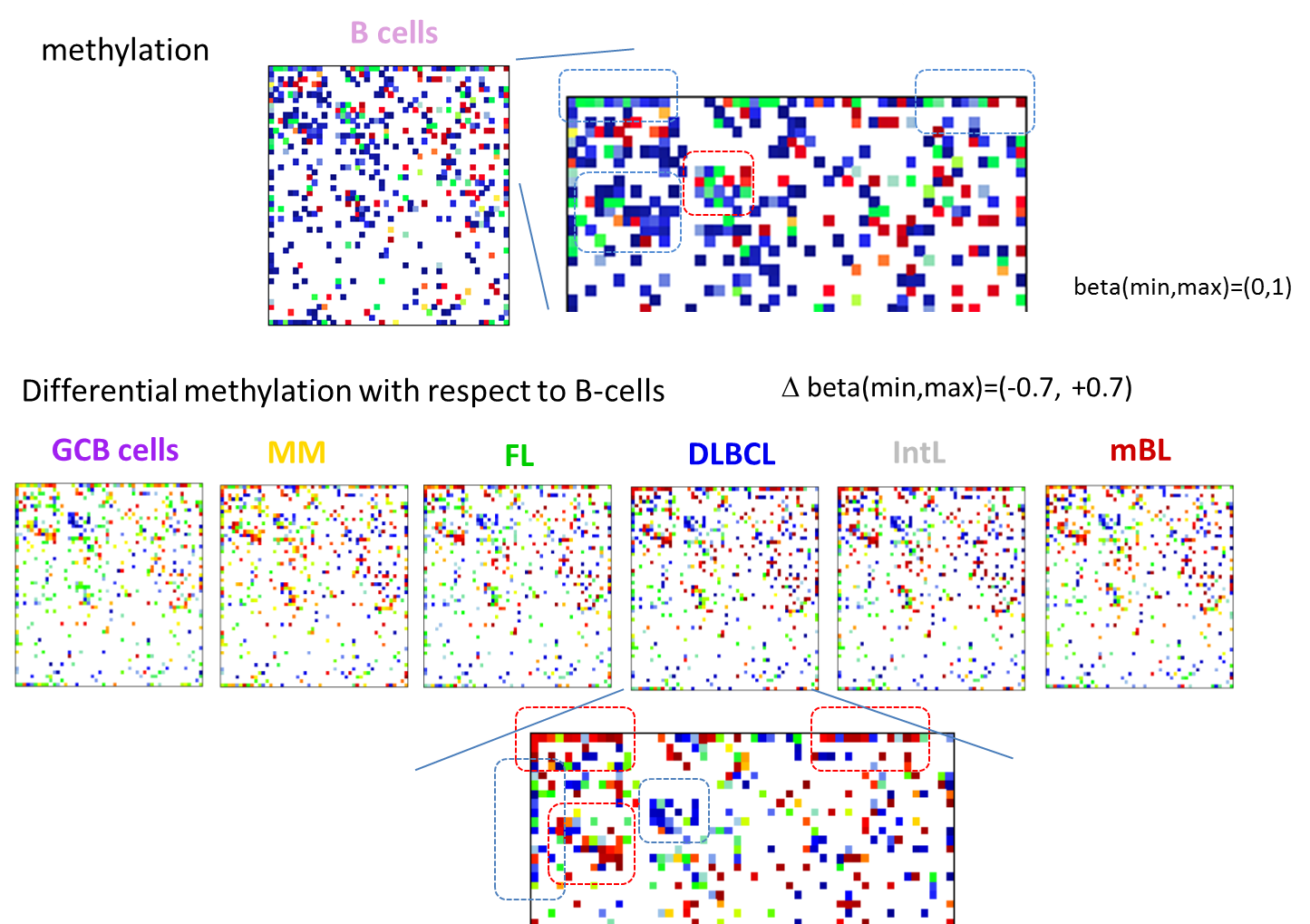


**Figure S3.** Mapping of methylation levels of genes probed by the methylation arrays into the expression SOM. Note that only about 800 of the 22,000 genes considered in the expression arrays were on the methylation arrays. Beta values were shown for B cells (blue tiles refers to beta = 0 and red to beta = 1 and empty/white tiles to unpopulated metagenes). For the other classes delta beta values with respect to B cells were shown. No clear accumulation of high and low beta/delta beta values within larger areas of the maps can be detected. Enlargement of the maps for B cells however reveals local accumulations of high and low methylation levels, where the former tend to become hypo- and the latter to become hypermethylated (see map for DLBCL). The map thus resolves the global trend illustrated in Figure 1 on metagene and spot levels.


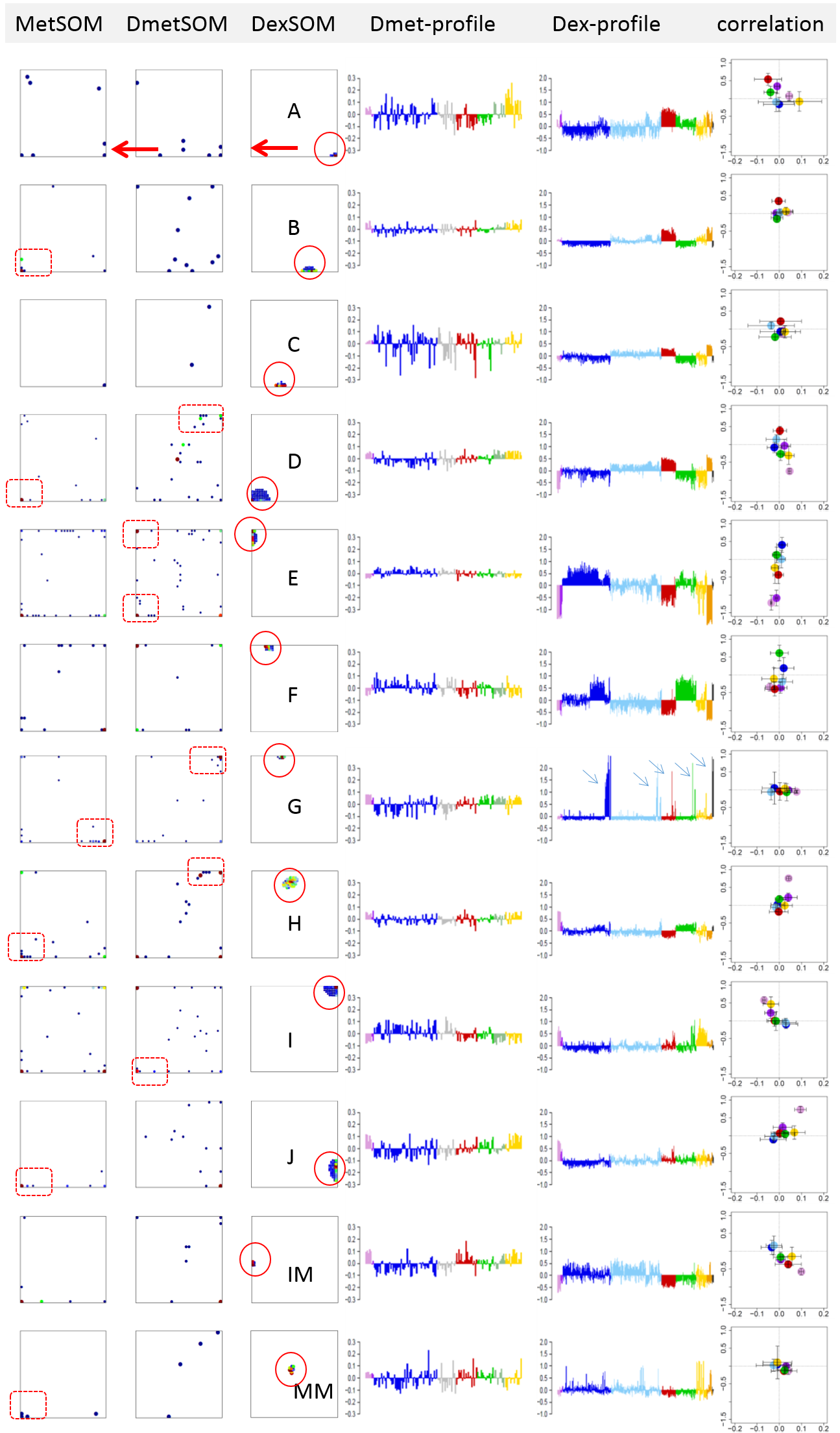


**Figure S4.** *Cont.*


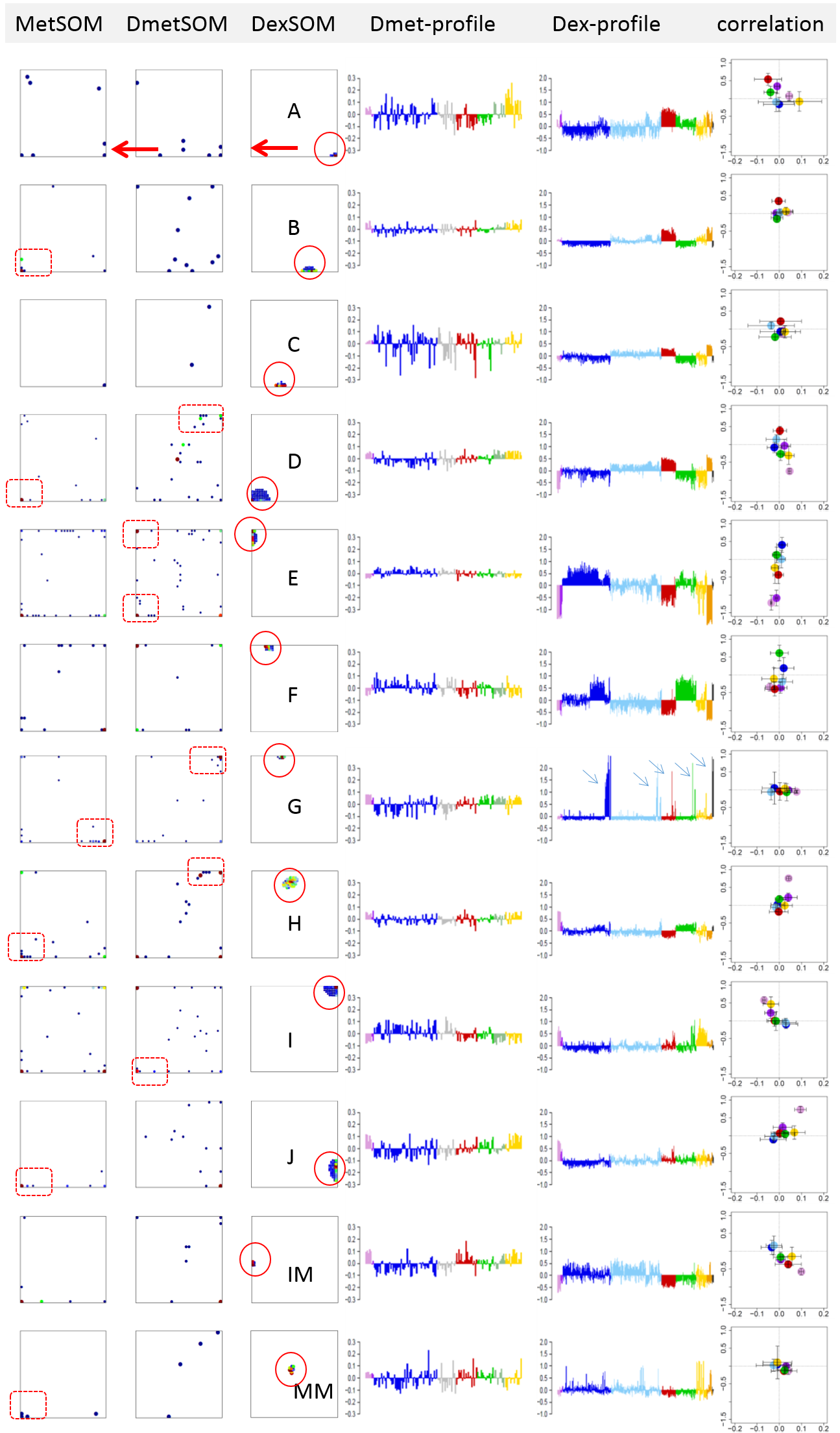

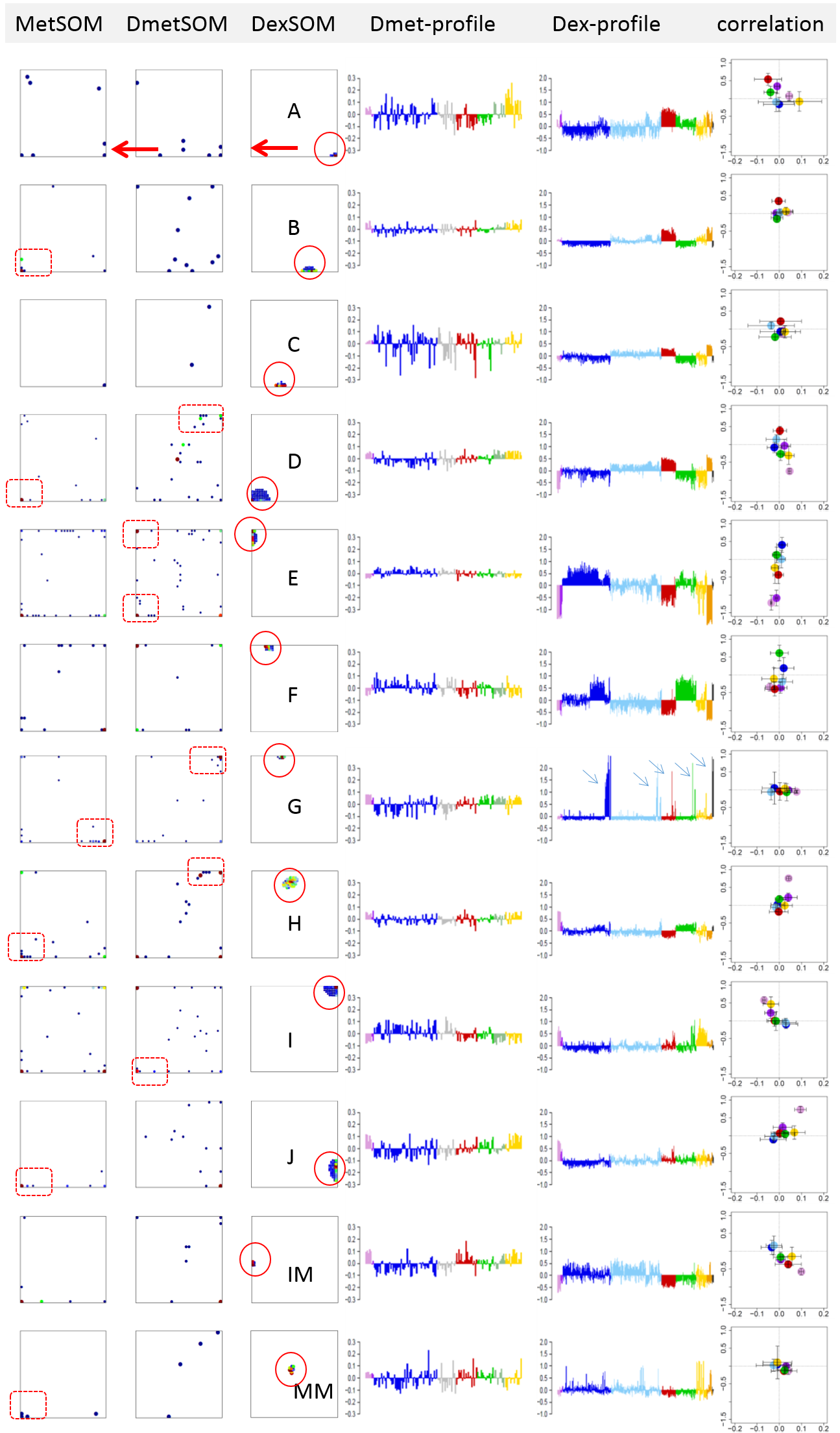


**Figure S4.** Mapping of genes from the expression spots A – J, IM, MM (see Figure 6) into the methylation SOM. See description of Figure 8 for assignments. Most of the Dex-spots show only weak correlation with the methylation of the respective genes: In the methylation SOM the genes show only a diffuse distribution without strong accumulation in distinct regions. This result seems reasonable if one assumes that alterations of methylation cause alterations of gene expression (and not vice versa) and that concerted expression is induced also by effects not related to methylation (e.g., by gene regulatory networks acting via transcription factors). In this case co-methylation effects will be “diluted” in expression data and co-expression clusters will map only partly into methylation data. On the other hand, part of the expression clusters nevertheless correlate with the methylation data. Strong downregulation of expression in lymphoma associates with hyper- (spot I) and hypo- (H, J) methylation and also up-regulation in lymphoma with hypo- (A, D, IM) methylation. The profiles of the tonsil-related spot G show that progressive hypo-methylation correlates with up-regulated gene expression of the respective lymphoma samples presumably contaminated with healthy tonsil tissue (see arrows).


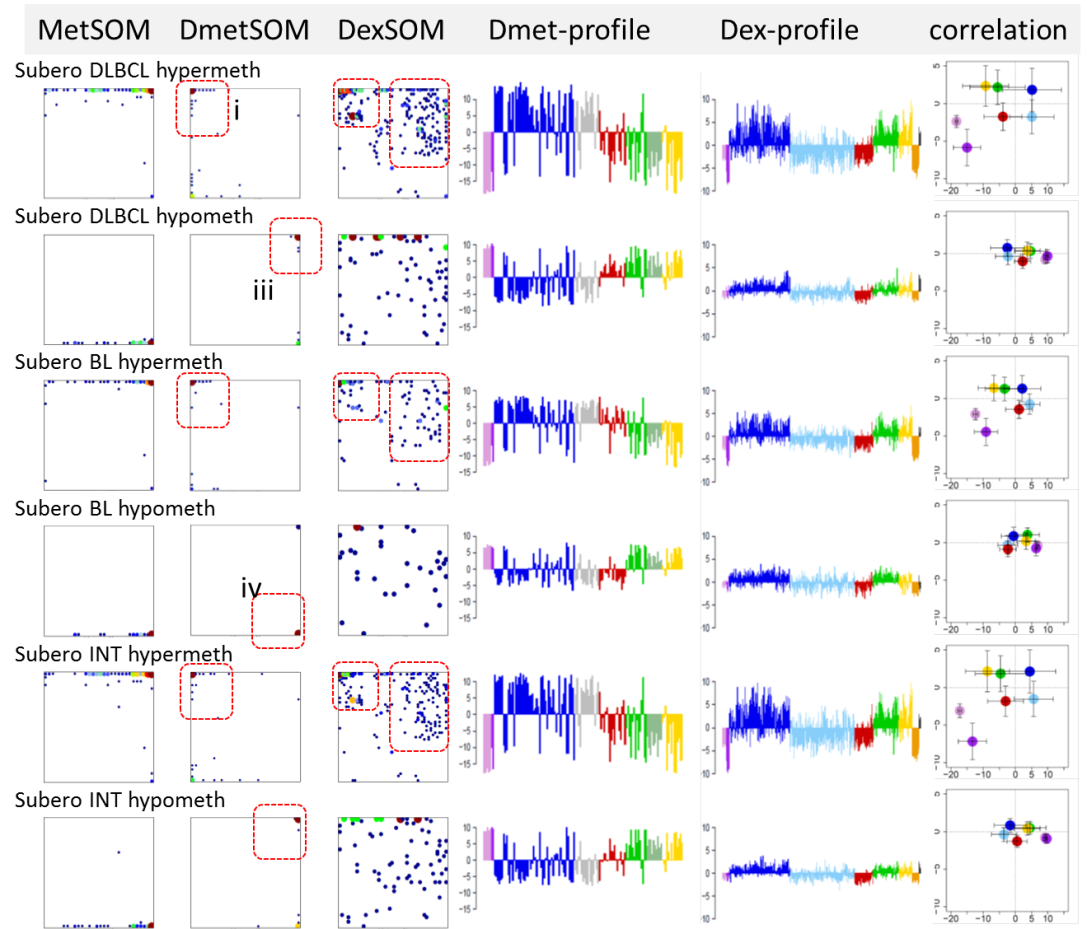


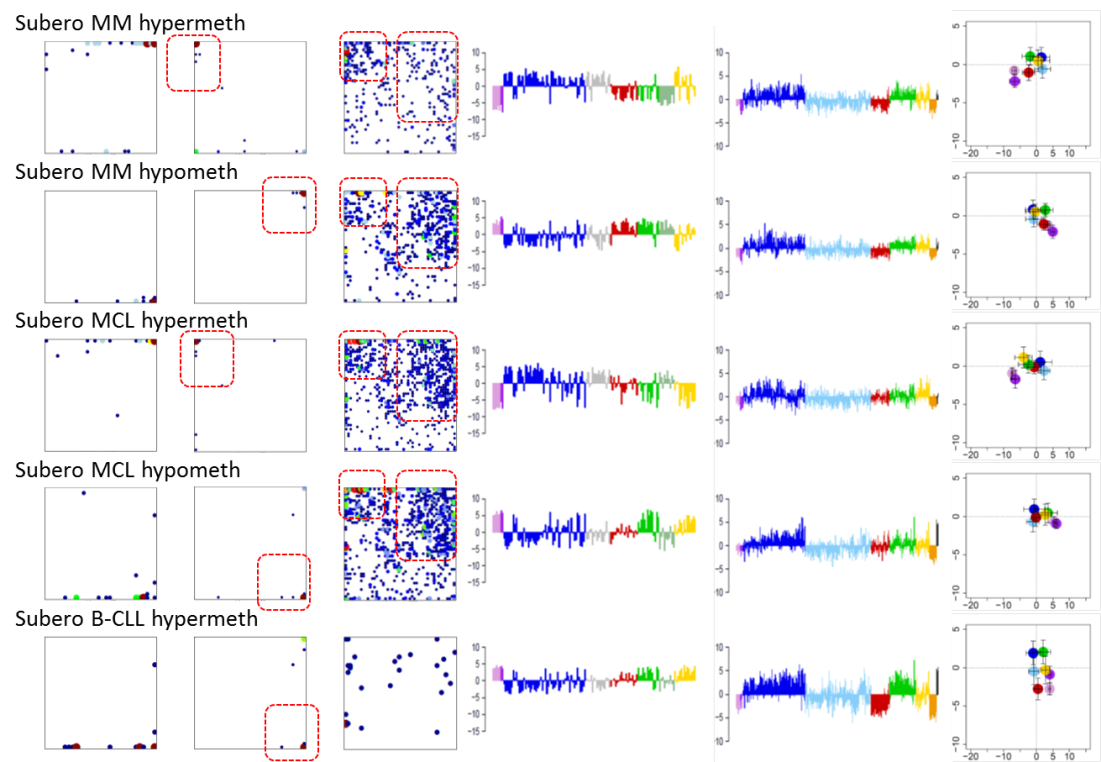


**Figure S5.** Mapping of genes differentially methylated in the histological lymphoma classes with respect to B-cells as determined in [1]. Note that hyper-methylated genes in DLBC, IntL, BL, FL and partly MCL show very similar characteristics agreeing also with that of genes of Dmet-spot i (see Figure 7). The same behavior was found for hypo-methylated genes in these lymphomas which mostly accumulate in Dmet-spot iii. Note that the maps of hyper-methylated genes for MM, FL, IML and mBL show the same characteristics as for DLBCL despite the smaller number of genes detected and the smaller total methylation effect. Interestingly, also gene sets functionally related to “extracellular space”, hyper-methylated in glioblastoma referring to the CpG-island methylator phenotype (GCIMP) and “G-protein coupled receptor activity” occupy similar regions in the SOM and show nearly identical expression profiles in the subtypes studied.


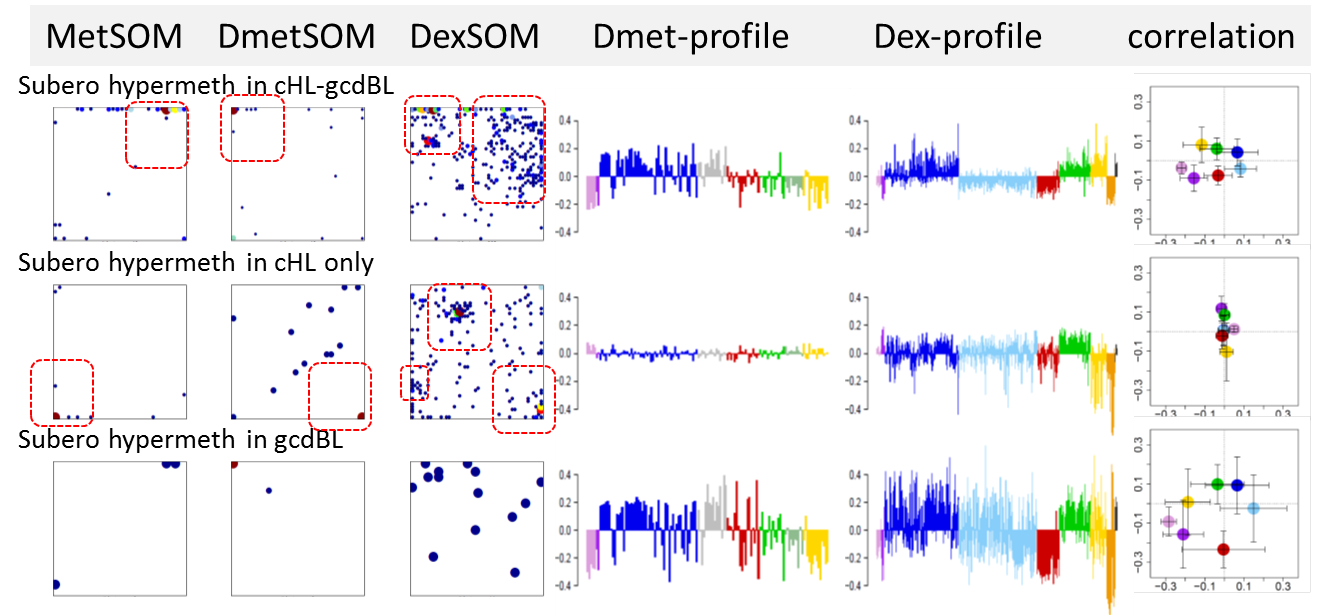


**Figure S6.** Mapping of genes hyper-methylated in classical Hodkin lymphoma (cHL) and/or in GC derived B cell lymphoma (gcdBL) with respect to B cells as determined in [2]. Genes hypermethylated in both, cHL and gcdBL, agree in their characteristics with that hypermethylated in our study, as expected. Interestingly, genes specifically hypermethylated in cHL only accumulate in three regions of the DexSOM referring to two spots H and J related to healthy B cell functions showing downregulated gene expression in lymphoma, especially in BCL. A third spot of accumulation (spot IM) is related to the activation NFκB-pathway, a molecular hallmark of cHL. Note also that the tiny effect of differential methylation between all lymphoma classes and references studied. Hence, cHL shows the methylation and expression characteristics of gcBL but in addition a specific methylation profile related to the molecular hallmarks of this cancer.


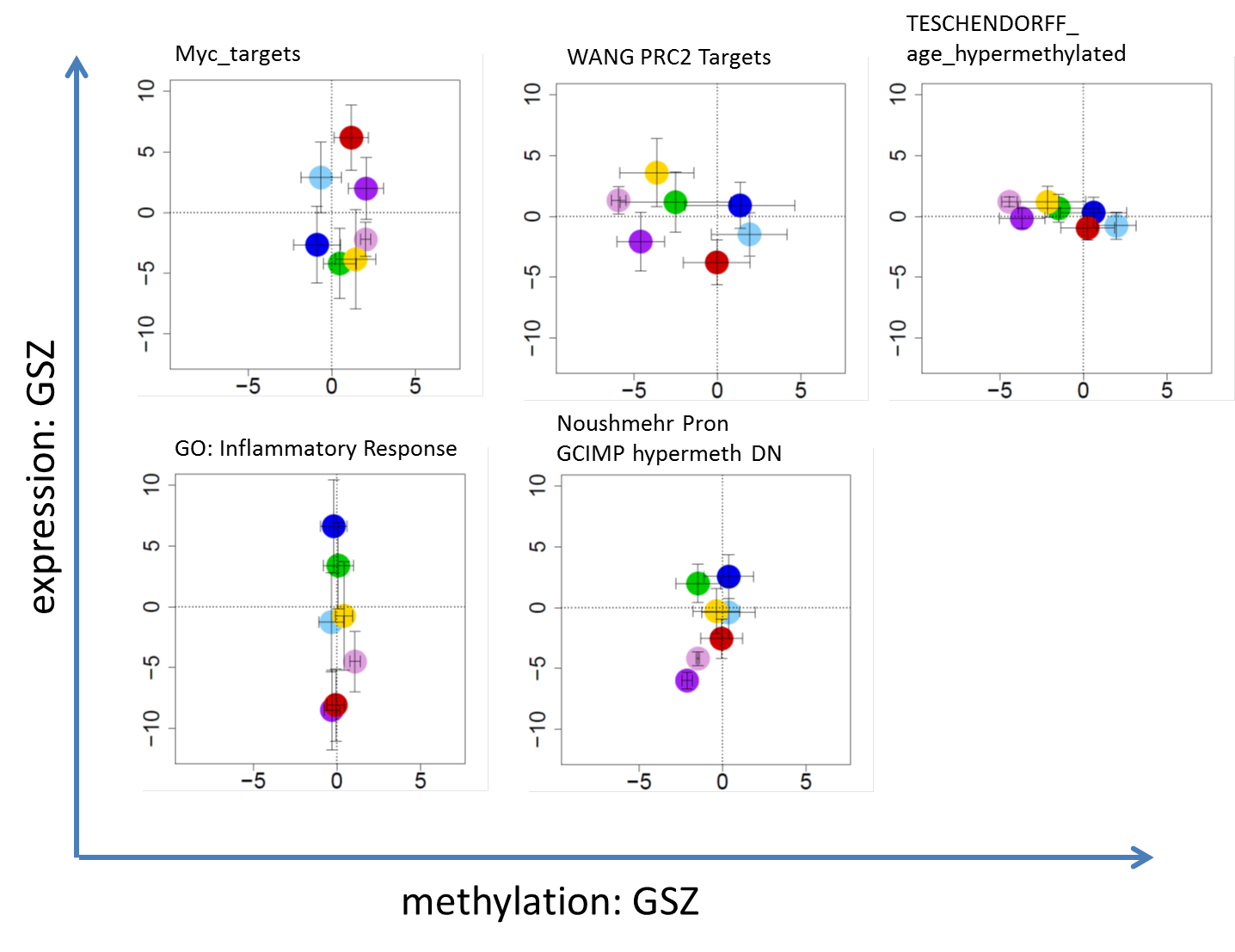


**Figure S7.** Correlation plots between Dmet and Dex GSZ-data of selected gene sets enlarged from Figure 8.


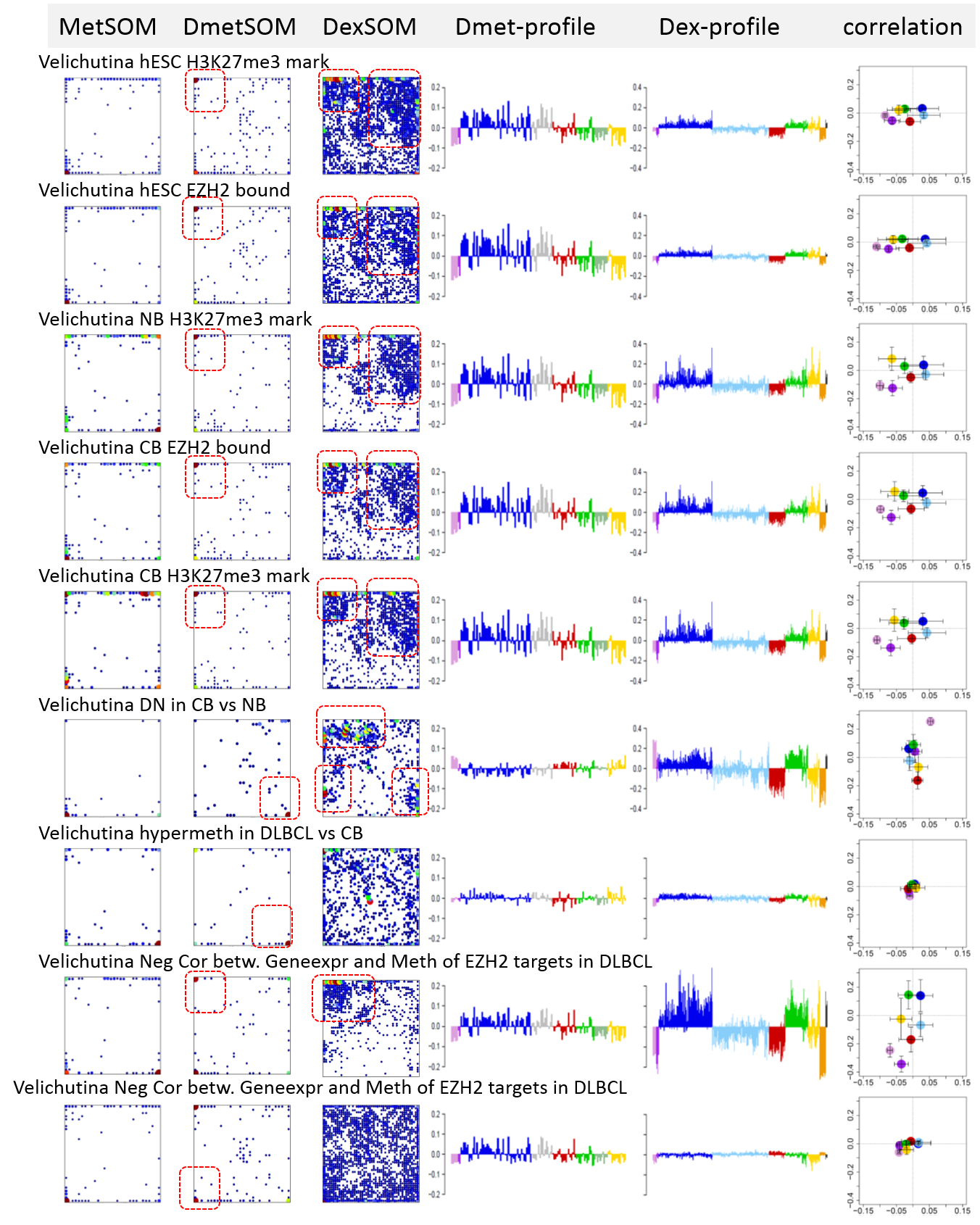

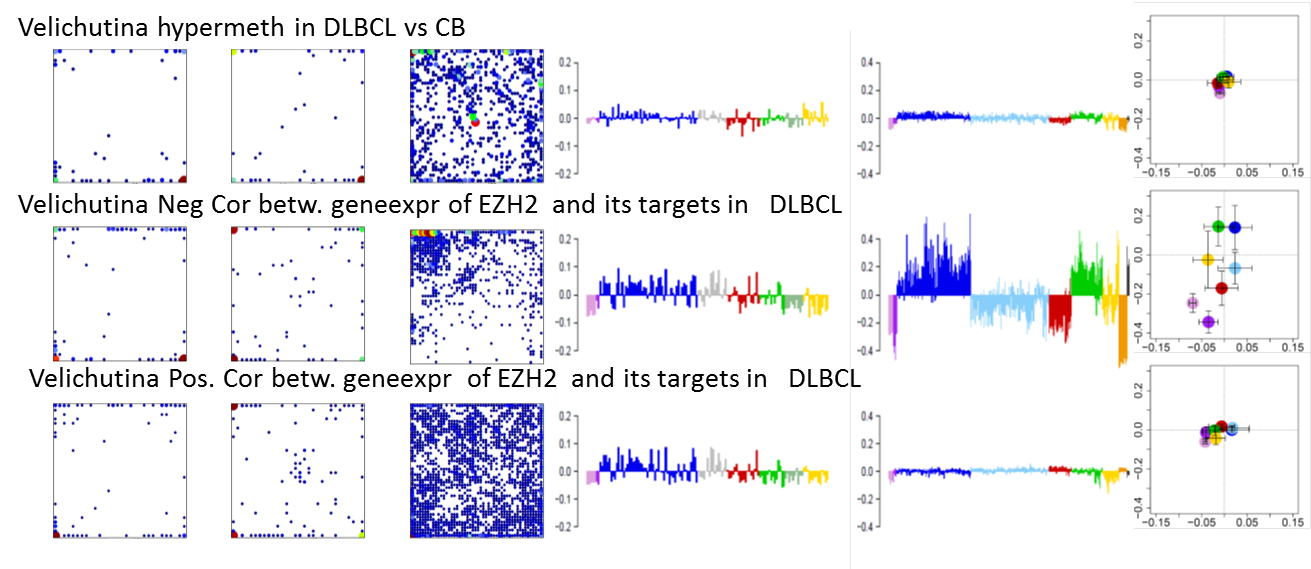


**Figure S8.** Characteristics of genes marked by H3K27me3 and/or representing targets of *EZH2* in human embryonal stem cells (hESC), naïve B cells (NB, only H3K27me3) and centroblasts (CB); de-novo *EZH2* targets differently expressed between CB and NB, hypermethylated in DLBC compared with CB and showing negative and positive correlation between *EZH2* gene expression and *EZH2*-targets. Gene sets were taken from [3].


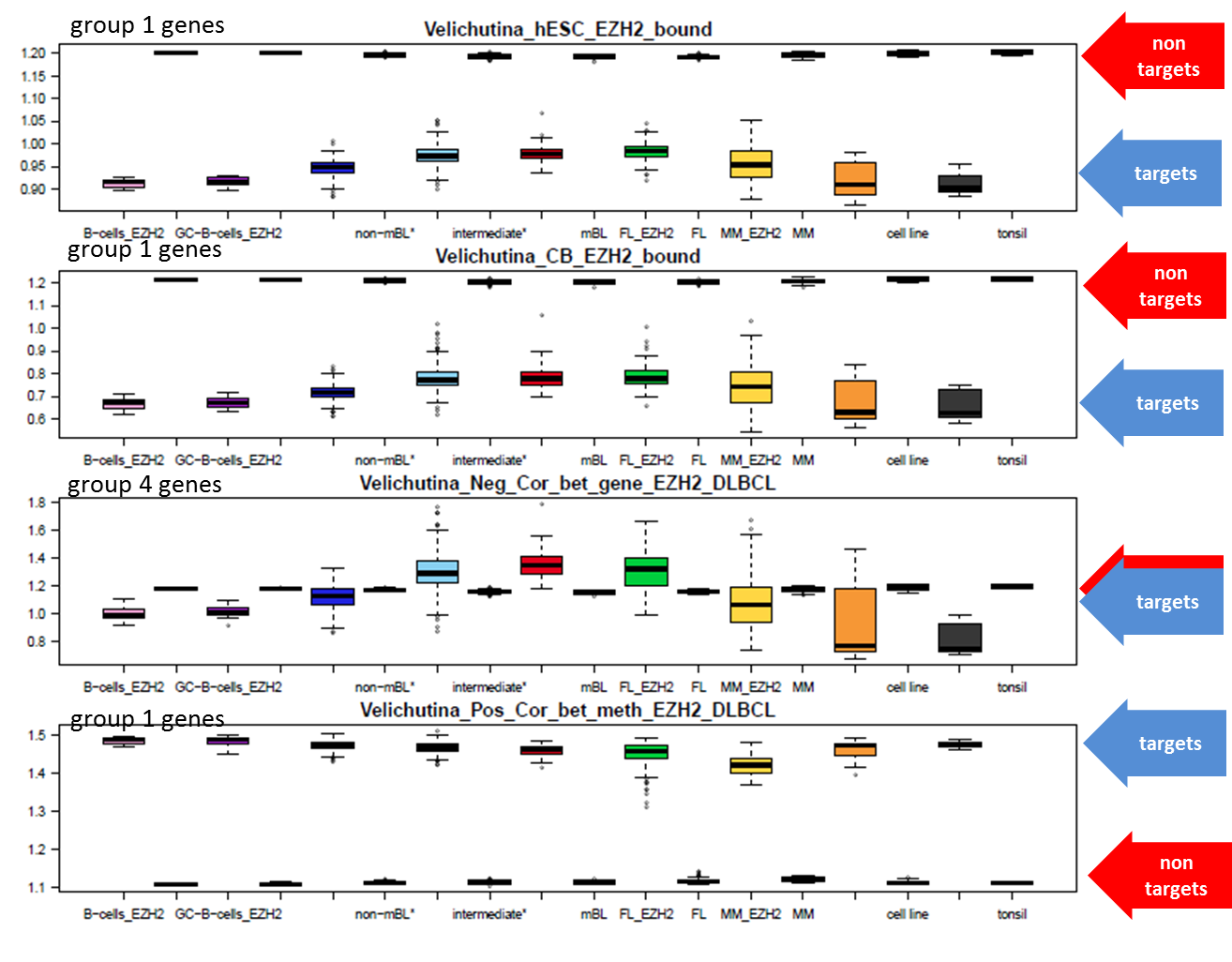


**Figure S9.** Comparison of absolute gene expression levels between *EZH2* targets and
*EZH2*-non targets. Gene sets of *EZH2* targets (see also Figure S8) were taken from [3]. Gene expression data were quantile normalized but not centralized. Mean gene expression values log-averaged over the respective selection of genes was calculated for each sample and then shown a boxplot for all samples of a given class. For all EZH2 targets one finds reduced expression compared with non-targets in CB, NB and lymphoma in agreement with [3]. The degree of correlation between *EZH2* expression and the expression of its targets affects the total expression level: The mean expression of all *EZH2*-target is smallest, comparable with the expression level of non-targets. Both expression levels are comparable for *EZH2* targets the expression of which changes in anti-concert with that of the *EZH2* gene and expression level of *EZH2* targets even markedly exceed that of non-targets for targets the expression of which changes in concert with that of the *EZH2* gene. Inspection of the location of the respective genes in the DexSOM in Figure S8 shows that the different sets can be assigned to group 1, group 2 and group 4 genes as discussed in the main paper and indicated in the figure with total expressions group 3 < group 1 < group 4 < group 2.


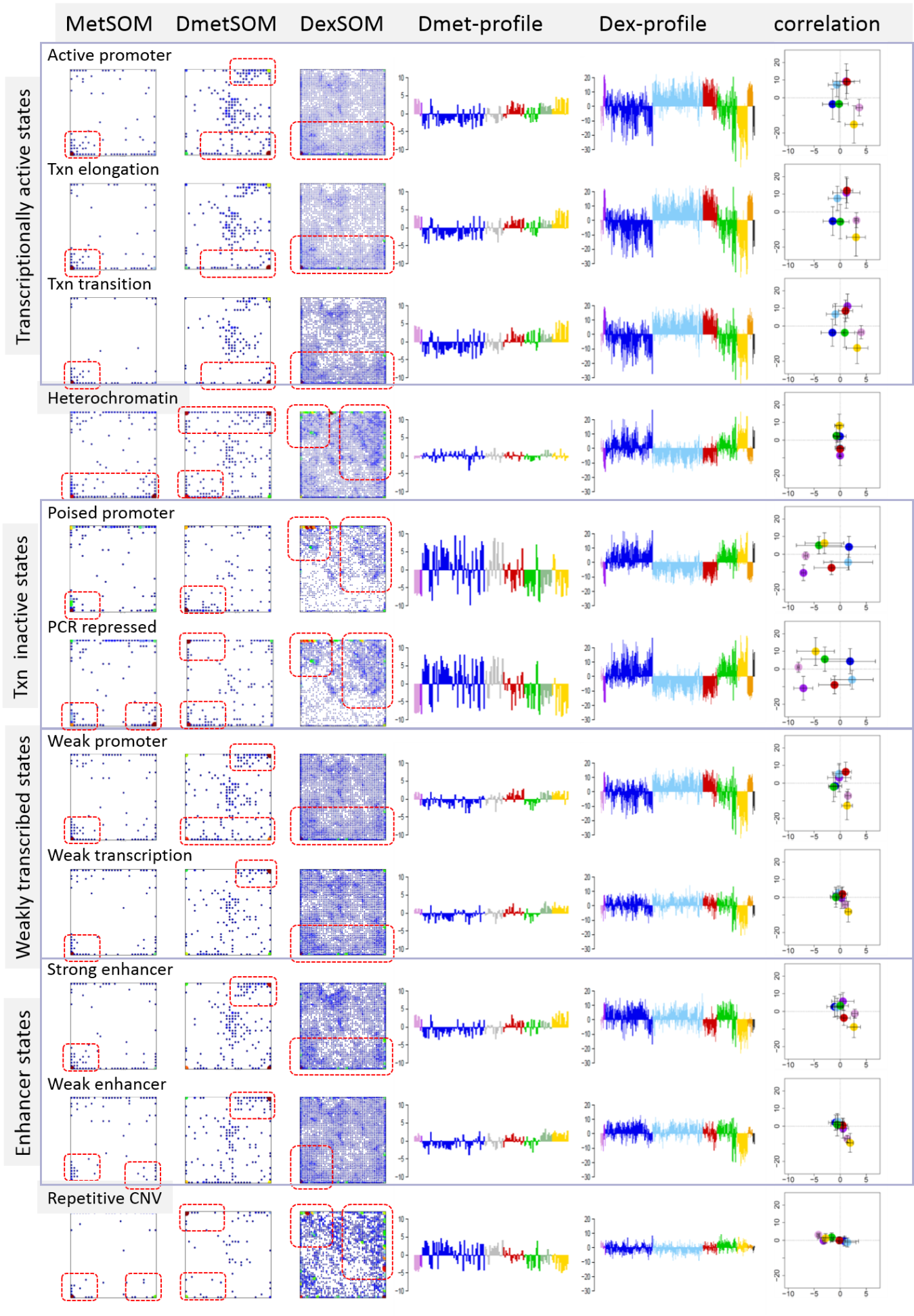


**Figure S10.** Mapping of genes referring to different chromatin states as determined in lymphoblastoid cells using ChIP-Seq and a Hidden Markov analysis [4,5]. Each chromatin state comprises from 1875 to 9946 genes which are further analyzed as gene set as described above. According to the methylation and expression characteristics in the lymphoma data set these gene sets can be grouped into five types as indicated in the figure.

References

1. Martin-Subero, J.I.; Ammerpohl, O.; Bibikova, M.; Wickham-Garcia, E.; Agirre, X.; Alvarez, S.; Brüggemann, M.; Bug, S.; Calasanz, M.J.; Deckert, M.; *et al*. A comprehensive microarray-based DNA methylation study of 367 hematological neoplasms. *PLoS ONE* **2009**, *4*, e6986.
2. Martín-Subero, J.I.; Kreuz, M.; Bibikova, M.; Bentink, S.; Ammerpohl, O.; Wickham-Garcia, E.; Rosolowski, M.; Richter, J.; Lopez-Serra, L.; Ballestar, E.; *et al*. New insights into the biology and origin of mature aggressive B-cell lymphomas by combined epigenomic, genomic, and transcriptional profiling. *Blood* **2009**, *113*, 2488–2497.
3. Velichutina, I.; Shaknovich, R.; Geng, H.; Johnson, N.A.; Gascoyne, R.D.; Melnick, A.M.; Elemento, O. EZH2-mediated epigenetic silencing in germinal center B cells contributes to proliferation and lymphomagenesis. *Blood* **2010**, *116*, 5247–5255.
4. Ernst, J.; Kheradpour, P.; Mikkelsen, T.S.; Shoresh, N.; Ward, L.D.; Epstein, C.B.; Zhang, X.;
   Wang, L.; Issner, R.; Coyne, M.; *et al*. Mapping and analysis of chromatin state dynamics in nine human cell types. *Nature* **2011**, *473*, 43–49.
5. Ernst, J.; Kellis, M. Discovery and characterization of chromatin states for systematic annotation of the human genome. *Nat. Biotech.* **2010**, *28*, 817–825.
